# The architecture of the *Plasmodiophora brassicae* nuclear and mitochondrial genomes

**DOI:** 10.1038/s41598-019-52274-7

**Published:** 2019-10-31

**Authors:** Suzana Stjelja, Johan Fogelqvist, Christian Tellgren-Roth, Christina Dixelius

**Affiliations:** 1 Department of Plant Biology, Uppsala BioCenter, Linnéan Center for Plant Biology, Swedish University of Agricultural Sciences, P.O. Box 7080, SE-75007 Uppsala, Sweden; 2 Uppsala Genome Center, Science for Life Laboratory, Department of Immunology, Genetics and Pathology, Uppsala University, BMC, Box 815, SE-751 08 Uppsala, Sweden

**Keywords:** Evolution, Plant sciences, Microbiology

## Abstract

*Plasmodiophora brassicae* is a soil-borne pathogen that attacks roots of cruciferous plants causing clubroot disease. The pathogen belongs to the Plasmodiophorida order in Phytomyxea. Here we used long-read SMRT technology to clarify the *P. brassicae* e3 genomic constituents along with comparative and phylogenetic analyses. Twenty contigs representing the nuclear genome and one mitochondrial (mt) contig were generated, together comprising 25.1 Mbp. Thirteen of the 20 nuclear contigs represented chromosomes from telomere to telomere characterized by [TTTTAGGG] sequences. Seven active gene candidates encoding synaptonemal complex-associated and meiotic-related protein homologs were identified, a finding that argues for possible genetic recombination events. The circular mt genome is large (114,663 bp), gene dense and intron rich. It shares high synteny with the mt genome of *Spongospora subterranea*, except in a unique 12 kb region delimited by shifts in GC content and containing tandem minisatellite- and microsatellite repeats with partially palindromic sequences. *De novo* annotation identified 32 protein-coding genes, 28 structural RNA genes and 19 ORFs. ORFs predicted in the repeat-rich region showed similarities to diverse organisms suggesting possible evolutionary connections. The data generated here form a refined platform for the next step involving functional analysis, all to clarify the complex biology of *P. brassicae*.

## Introduction

Unicellular eukaryotes or protists can be found in any habitat worldwide and fossil records are available for certain taxa where some are dated as early as the Precambrian period^1^. Among the protists, Rhizaria is a large and diverse organism group in the kingdom Chromista which has experienced a number of taxonomic re-evaluations^2^. A few plant pathogens can be found here among which *Plasmodiophora brassicae* is the most well known, located in the Phytomyxea class^3^. This plasmophorid pathogen attacks roots of numerous cruciferous plant species resulting in typical swollen roots (clubs or galls), giving rise to the disease name. The clubroot disease has been known about 100 years, and is now present in more than 60 countries worldwide^4–6^. *P. brassicae* is an obligate biotroph and has a strict requirement of host tissue for growth and multiplication. The resting spores of *P*. *brassicae* can survive in soil for many years, before hatching under suitable conditions followed by zoospore release. The host plant infection process is divided into two phases. A short stage where root hairs are infected by zoospores in the soil, followed by formation of primary plasmodia and second round of zoospores. Upon release, these zoospores, initiate a new round of infection where large intracellular plasmodia in root cortical cells develops^7,8^. Genetic recombination is thought to take place during zoospore development and early infection phases but these events and possible reproduction stages are not completely understood^9^. Further details on biology and other aspects on *P. brassicae* are covered elsewhere^10–12^.

Very restricted genome information is available from organisms in Rhizaria. This is because they commonly colonize complex ecological niches from which pure DNA is difficult to retrieve in required quantities. Besides *P. brassicae*, nuclear data today derive from the foraminifera *Reticulomyxa filosa*, the chlorarachniophyte alga *Bigelowiella natans*, the plasmophorid *Spongospora subterranea* and transcriptome datasets on marine species^13–18^. *S*. *subterranea* is another soil-borne pathogen causing the potato powdery scab disease but this organism can also act as a vector for the Potato Mop Top Virus^19^. *P. brassicae* and *S*. *subterranea* are the two most closely related plasmodiophorids based on present sequence information^20^.

Application of long-read sequencing greatly supports not only reliable pathotyping (diagnostics) or identification of important structural variants when genomic gaps with repetitive or unique sequences are closed but also accurately resolve chromosomes in complex genomes. Genomic information of high quality is crucial for enhancing our understanding of important evolutionary events, which could give us insights into related organisms together with events behind lost and gained traits. Such information could in case of plant pathogens be extra valuable for development of durable control strategies. Reliable genomic information is essential not least for experimentally challenging obligate biotrophic soil-borne pathogen as *P. brassicae* where a number of questions in its life-cycle and differences between pathotypes remain to be clarified.

We previously made a *de novo* genome assembly of the *P. brassicae* strain e3 based on a combination of Illumina and 454 sequencing approaches^16^. This achievement was followed by genome information for isolates from Canada, China and Germany based on similar technologies^21–23^. The former sequencing of the *P. brassicae* e3 genome^16^ generated two mitochondrial contigs whose complete assembly could not be achieved, thus the mitochondrial sequence was not made public. Here, we applied long-read PacBio RSII single molecule real-time (SMRT) sequencing technology to fill in sequence gaps and further resolve regions with repetitive sequences of the *P. brassicae* e3 genome. We present new data based on twenty contigs representing the nuclear genomic content of *P. brassicae* e3 and one contig covering the entire mitochondrial genome generated by this approach. We were able to identify telomere sequences similar to those found in green algae and *Theileria annulata*, an apicomplexan parasite. The assembly and *de-novo* annotation of the mitochondrial genome revealed a 114,663 bp large genome with a complex sequence organization and a distinct 12 kb repeat-rich region. These findings emphasize the value of long-reads in resolving unrecognized genomic variation and highlight the importance of distinguishing biological from technical sequence differences.

## Results and Discussion

### *P. brassicae* has a T_4_AG_3_ telomere repeat composition and possess meiosis-related proteins in the nuclear genome

**Figure 1:**
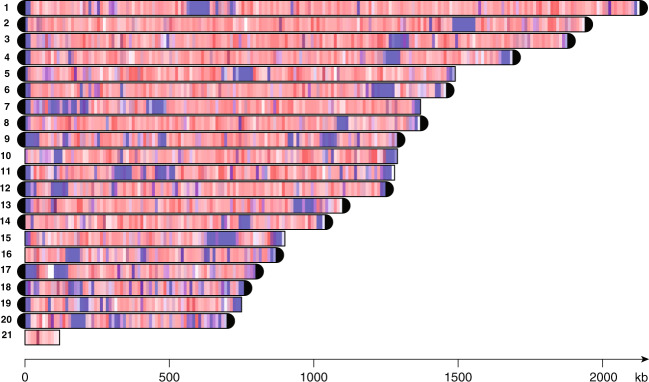
Contigs and telomeres in *Plasmodiophora brassicae* e3 strain. Contig sizes of the nuclear (no. 1 to 20) and mitochondrial genome (no. 21). Telomeres are marked with black ends. Density of coding (red) and repeat (blue) sequences in non-overlapping sliding 10 kbp windows. The color intensity is proportional to the given feature density.

By using the long-read SMRT technology, the numbers of nuclear assembled contigs were reduced from 165^16^ to 20 (Supplementary Table S1). In the 25 Mb nuclear genome coding sequences were evenly distributed on each scaffold, interspersed with minor repeat regions (Fig. 1). Repetitive sequences were looked for manually and by using the tandem repeats finder^24^. Centromeric candidates with different length could be found on all nuclear contigs (Fig. 1). Thirteen of the 20 contigs of the nuclear genome (ranging from sizes between 692,149 bp to 2,120,846 bp) represent complete chromosomes from telomere to telomere (Fig. 1; Supplementary Table S2). The [TTTTAGGG] or T_4_AG_3_ telomeric sequences of *P. brassicae* (Supplementary Fig. S1) are identical with those found in *Chlamydomonas reinhardtii*, the green alga^25^ and *Theileria annulata*, an apicomplexan animal parasite^26^. Among Archaeplastida, the T_3_AG_3_ telomeric motif is most common in plants for example in the *P*. *brassicae* e3 host *Arabidopsis thaliana* whereas the telomeres in red and green algae vary between T_4_AG_3_, T_3_AG_3_, T_2_AG_3_, and T_2_AG_6_^27,28^. Five of the remaining seven *P. brassicae* contigs were terminated by a single telomere. Efforts to further assemble all the remaining sequences into longer contigs terminated with telomeres were not successful. This could be explained by the enrichment of repetitive sequences at the ends making overlapping *in silico* analysis and additional PCR analysis followed by Sanger sequencing unsuccessful. Alternatively, the seven contigs may be dispensable or form supernumerary chromosomes. The organization of the nuclear chromosomes into two groups: a core set (permanent chromosomes) and accessory, dispensable, or supernumerary chromosomes is common among plant pathogenic fungi^29^. The extra chromosomes are known to play important roles for evolution, high recombination rates and adaptation to external changes^30^. Commonly the dispensable chromosomes are enriched for genes involved in host colonization, infection and traits attributed to polymorphism observed between different isolates. Any such information and connection is yet not reported in *P. brassicae*. Earlier analyses based on pulse-field gel electrophoresis revealed between 6 to 16 chromosomal bands ranging from 680 kb to 2.2 Mb in size, including chromosomal polymorphisms between different single-spore and field isolates^12,31^. Whether the different observations reflect biological differences or are technical related remains to be clarified.

The MAKER^32^ annotation pipeline combined with several *ab-initio* gene predictors generated 9,231 protein-encoding genes in the *P. brassicae* nuclear genome (Supplementary Table S1). In a comparison of *P. brassicae* with *B. natans, R. filosa* and the transcriptome of *S. subterranea* 5,605 proteins were earlier assigned to be *P. brassicae* specific^16^. In an extended and new comparison including additional transcriptome data from marine species^15^ a joint core of 476 proteins shared with 12 other Rhizaria species was found. This revised analysis generated 3,017 *P. brassicae*-specific proteins whereof a majority lacked functional annotations (Supplementary Fig. S2; Supplementary Table S3).

Synaptonemal complexes (SC) were found by using serial thin sections of *P. brassicae* for electron microscopy^33^. SC, a proteinaceous structure, is known to assemble at the interface between pairs of homologous chromosomes at prophase I (zygotene) of meiosis I. SC formation is an essential feature of meiosis but is not strictly conserved in all organisms^34–36^. We used our new *P. brassicae* genome sequence to search for SC-associated and meiotic-related gene information (Supplementary Table S4) and next monitored their activity in enriched life stages of *P. brassicae* (Supplementary Fig. S3). Candidates such as *HOP1, ZIP1, ASY2, REC8, MER3, MSH5, FKBP6* were found to be highly active whereas animal orthologs such as C(3)G, SYCP1 and SYP2 were suppressed. SC proteins and important proteins for SC modification are known to vary on sequence level^36,37^ which implies that additional genes with similar functions but not identified here can be present in *P. brassicae*. However, much remains to be learnt on SC components, their dynamics and regulation in general, and not least in *P. brassicae*.

### *P. brassicae* has a large mitochondrial genome with a 12 kb repeat-rich region

The mitochondrial genome represented by a single contig has a circular structure and a size of 114,663 bp (Supplementary Table S2). A uniform coverage with an average depth of ~800x was obtained across the genome, except from 47,000 to 50,000 bp where the depth decreased to ~400×. The possibility of miss-assembly was minimized by well-aligned reads spanning over this 3 kb stretch of AT-rich sequences that caused the reduced depth. The AT-rich sequences were found to be part of a 12,500 bp long repeat-rich region (42,650–55,150 bp) by a dot-plot self-similarity comparison (Supplementary Fig. S4a). A closer look into the dot plot indicated presence of tandem minisatellite- and microsatellite repeats with partially palindromic sequences in this region (Supplementary Fig. S4b).

Mt sequences are available for three *P. brassicae* strains: Pb3 from Canada^21^, ZJ-1 from China^22^ and eH from Germany^23^. In BLASTn search of the whole-genome shotgun database the mt sequence of e3 shared high identity (>99.95%) with these strains (Supplementary Table S5). However, the e3 mt sequence is about 11, 13 and 21 kb longer than eH, Pb3 and ZJ-1, respectively. We next compared the mt sequences from these three *P. brassicae* strains with e3, here visualized by dot-plots. While the sequences show high similarity for most of their length, the 12 kb repeat-rich region in e3 was not resolved in any other strain (Supplementary Figs. S5–S7). Neither was the repeat-rich region identified in the mt contigs we generated earlier by applying Illumina/454 technologies on the e3 genome^16^. The previously excluded sequence is provided here (Supplementary Table S6). The difference between the updated and former e3 mt sequence is visualized in Supplementary Fig. S8.

**Figure 2:**
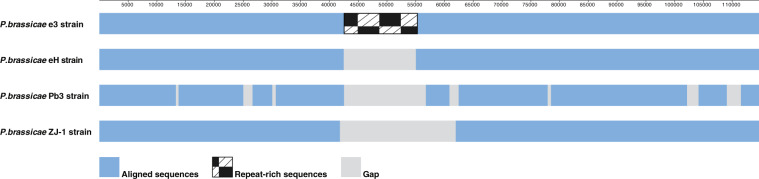
Alignment between four *Plasmodiophora brassicae* mitochondrial genomes. The illustration is based on mitochondrial genome synteny using the e3 strain (114,663 bp) as a template to which homologous regions identified in eH (102,962 bp), Pb3 (101,103 bp) and the ZJ-1 (93,640 bp) strain were aligned. For details, see Supplementary Fig. S9. The upper line represents sequence coordinates of the e3 mitochondrial genome.

When analyzing mitochondrial synteny between the four *P. brassicae* strains, two locally collinear blocks (LCB) with highly conserved sequences and no rearrangements were identified by the whole genome alignment tool Mauve^38^ (Supplementary Fig. S9). The area outside LCBs corresponds to the repeat-rich region identified in the e3 genome and lacked detectable homology in the other three genomes. If the homologous LCBs from eH, Pb3 and ZJ-1 are aligned to the e3 mt genome as illustrated in Fig. 2, absence of the repeat-rich region considerably contributes to genome size differences observed between the strains.

In conclusion, the mt intragenomic variation observed between the four strains (Fig. 2) is most likely technical rather than biological related. Several studies have reported unrecognized variation by short-read sequencing technologies and demonstrated that long reads which can span highly repetitive regions and thereby facilitate assembly, are essential for correct genome resolution^39,40^.

### The *P. brassicae* mt genome has a complex gene organization

We initially annotated the *P. brassicae* e3 mt sequence with MFannot, an automated tool commonly used for gene prediction in organelle genomes^41^. However, in the MFannot output many protein-coding and RNA genes were fragmented and with numerous introns predicted in intergenic regions, indicating interruption of coding sequences and incomplete annotations. Similar “mosaic” gene structure was reported in the eH mt genome annotated by MFannot^23^. To optimize *de novo* annotation, we combined automated predictions done with Prokka^42^ and MAKER2^43^ using Repeatmasker^44^, tRNAscan-SE^45^, Uniprot/Swiss-Prot mitochondrial proteins, the ribosomal database^46^ and Rfam^47^. *P. brassicae* e3 transcriptome data^16^ were re-assembled and mapped to the PacBio mt sequence to provide further information. The annotated mt genome of *S. subterranea*^48^ was used as an additional source. All data were uploaded to Web Appollo^49^, a web based annotation editing platform to facilitate manual curation.

**Figure 3:**
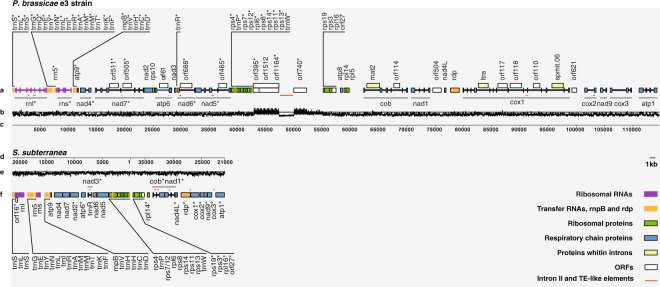
Physical maps and GC content of the *Plasmodiophora brassicae* e3 and *Spongospora subterranea* mitochondrial genomes. The two mitochondrial genomes have circular structure but are here linearized to facilitate comparisons. Boxes represent genes and exons separated by introns illustrated as lines. Colors correspond to specified gene functional groups. Genes transcribed from right to left have names marked with an asterisk. (**a**) Physical map of the *P. brassicae* e3 mitochondrial genome. (**b**) GC content of the *P. brassicae* e3 mitochondrial genome in non-overlapping sliding 20 bp windows. The horizontal line represents 50% GC. (**c**) Sequence coordinates of the *P. brassicae* e3 mitochondrial genome. (**d**) Sequence coordinates of the *S. subterranea* mitochondrial genome. For comparison, the *S. subterranea* sequence was opened at position 21,000 bp and two fragments (21,001-1 bp and 37,699–21,000 bp) were merged. (**e**) GC content of the *S. subterranea* mitochondrial genome in non-overlapping sliding 20 bp windows. The horizontal line represents 50% GC. (**f**) Physical map of the *S. subterranea* mitochondrial genome.

This extensive annotation procedure identified seventy-nine genes in the *P. brassicae* e3 mt genome. Thirty-two are predicted as protein-coding, 28 as structural RNA genes and 19 are ORFs (Fig. 3a; Supplementary Table S7). Seventeen protein-coding genes are involved in the mitochondrial respiratory chain, ten genes code for small ribosomal subunit proteins, four for proteins of the large ribosomal subunit and one gene (*rdp*) is coding for a RNA-directed DNA polymerase (Supplementary Table S8). Structural RNAs include large and small ribosomal subunits (*rnl* and *rns*), 5S ribosomal RNA (*rrn5*), a ribonuclease P type B (*rnpB*) and 24 transfer RNAs (Supplementary Table S9). By sequence similarity searches of the Uniprot/Swiss-Prot database (e-value < 10e-6) functional information was retrieved for ten ORFs (3 intergenic and 7 within introns) while nine ORFs (4 intergenic and 5 within introns) had no significant similarity (Supplementary Table S10). Seven intron group II and 2 transposon-like elements (Supplementary Table S11) and few simple repeats (Supplementary Table S12) were also found in the e3 mt genome.

In the *P. brassicae* eH strain sixty mt genes were identified^23^, divided on 32 protein-coding and 28 structural RNA genes corresponding to numbers and functional categories in e3. No transposable elements, ORFs, intron group II or repeats were reported. A detailed comparison of translated coding sequences could not be carried out here because amino acid sequences are not available for eH.

**Figure 4:**
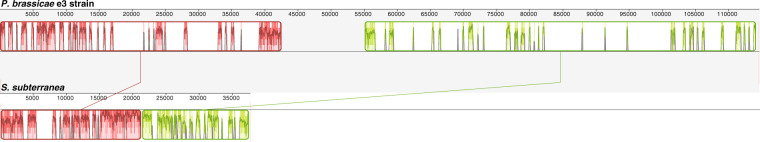
Synteny between the *Plasmodiophora brassicae* e3 and *Spongospora subterranea* mitochondrial genomes. The image^38^ illustrates aligned mitochondrial sequences of *P. brassicae* e3 (upper panel) and *S. subterranea* (lower panel). Identified homologous regions are displayed as two locally collinear blocks (LCB), green and red. The LCBs with matching colors between the genomes are connected by lines. Inside each LCB, a sequence similarity profile is shown, with its height representing the average level of sequence conservation. White areas within a LCB indicate sequences that are unique to a particular genome. The region outside LCBs (*P. brassicae* e3 sequence from 42,547 to 55,095 bp) has no detectable homology with the *S. subterranea* mitochondrial genome.

In comparison to its closely related potato scab pathogen *S. subterranea*^48^, the *P. brassicae* mt genome is roughly three times larger (Fig. 3a–f; Supplementary Table S7). When aligned, the genomes shared two syntenic blocks with well-conserved sequences, free from rearrangements (Fig. 4). Homology with the *S. subterranea* mt genome was not found in the 12 kb repeat-rich region in *P. brassicae* e3. Otherwise, the gene order between the genomes is nearly identical as shown in the gene maps (Fig. 3a,f). The map view demonstrates that presence of the repeat-rich region, high numbers of introns and variation in intergenic regions contributed to larger size of the *P. brassicae* e3 mt genome. Due to a larger mt genome size, *P. brassicae* e3 has a slightly lower coding density (54%) than *S. subterranea* (66%) (Supplementary Table S7).

When comparing the incidence of protein-coding genes, three genes (*atp8*, *rpl5* and *rps10*) were missing in the *S. subterranea* mt sequence (Supplementary Table S8). Several small ribosomal subunit proteins share overlapping sequences in the e3 mt genome, including *rps7/rps12* (19 bp), *rps11/rps13* (30 bp) and *rps3/rps19* (22 bp). Similar sequence overlaps between ribosomal genes are also seen in the *S. subterranea* mt genome^48^. The majority of e3 protein-coding genes (23 out of 32) start with the standard ATG codon (methionine) whereas TTA and TTG (leucine) seem to be used by five genes, and ATC and ATT (isoleucine) by three genes as alternative initiation codons (Supplementary Table S13). The GTG codon (valine) is employed only by *nad5*, in contrast to its extensive use among mt proteins in *Lotharella oceanica*, the rhizarian chlorarachniophyte alga^50^. Most of the e3 genes are terminated by TAA and in few cases with TAG codon. TGA, the third standard stop codon is translated into tryptophan in the mt genomes of *P. brassicae* e3 and *S. subterranea*^48^. This TGA usage pattern is therefore important to consider when selecting a correct genetic code for translation of *P. brassicae* mt coding sequences.

All structural RNA genes except arginine (*trnR*), proline (*trnP*) and tryptophan (*trnW*) were encoded on an 11,332 bp segment in the *P. brassicae* e3 mt genome (Fig. 3a). Densely located ribosomal RNA genes have been found in several other mt genomes and are believed to be involved in balanced co-transcription and RNA turnover^51^. Total number of tRNAs (24) was identical to that found in *S. subterranea*, with the difference that the e3 mt genome codes for glutamine (*trnQ*) and one copy of histidine (*trnH*) (Supplementary Table S9).

In contrast to *S. subterranea*, the *P. brassicae* e3 mt genome is strikingly intron-rich. Twelve genes in the e3 mitochondrial respiratory chain harbor the majority (41 out of 54) of the introns (Supplementary Table S8) while the remaining 13 introns are found in the large and small ribosomal subunit (Supplementary Table S9). Only five introns in three mt protein-coding genes were found in *S. subterranea*^48^, whereas chlorarachniophyte algae, *B. natans* and *L. oceanica* lack introns in their mt genomes^50^. Defining exon-intron splicing sites, especially in the genes with multiple exons was a challenge during annotation of the e3 mt genome and some positions remain to be exactly clarified. Application of the canonical acceptor-donor sites (GT-AG, GC-AG, AT-AC) resulted in reading frame shifts and disruptions of coding sequences predicted based on sequence alignments with known proteins. According to our current annotations supported by transcriptome data^16^, a new splicing pattern with the GT-AT and GA-CA acceptor-donor sites is likely employed by the *P. brassicae* e3 mt genome. Further, seven group II introns and two transposon-like elements, including Long Terminal Repeat (*LTR*) and Enhancer/Suppressor-mutator (*En/Spm*) were identified in the e3 mt genome (Fig. 3a; Supplementary Table S11). All these sequences except *En/Spm* were found to overlap exon-intron boundaries and may have an important role in the splicing mechanism. The MFannot tool^41^ predicted much higher number of group II introns (45) whereof 34 in gene regions (Supplementary Table S14). These introns were not submitted to the European Nucleotide Archive since no other annotation software supported their prediction. Annotated mt sequences from other plasmodiophorids are needed to resolve whether the higher number of group II introns represents an overestimated or a common landmark. Out of nineteen ORFs predicted in the *P. brassicae* e3 mt genome, the majority (12) was found within introns of the protein-coding genes (Supplementary Table S7). Seven of these ORFs showed significant similarity (e-value < 10e-6) with known proteins including among others three maturase-like proteins (*mat*1 and *mat*2), a group II intron-encoded protein (*LtrA*) and a DNA binding endonuclease (*spmit*.06) (Supplementary Table S10). Much remains to understand about evolution and regulatory function of the complex intron pattern in the *P. brassicae* e3 mt genome. It is intriguing to speculate that intron-encoded maturases could assist in the splicing processes, maybe promoting self-splicing as observed in fungal mitochondria^52^.

Overall GC content is close to identical in the mt genomes of *P. brassicae* e3 (26.2%) and *S. subterranea* (26.8%)^48^ while being lower compared to the chlorarachniophyte algae *B. natans* (42.2%) and *L. oceanica* (50.1%)^50^. However, large variation in the GC content was observed across the 12 kb repeat-rich region in *P. brassicae* e3 (Fig. 3b). A drastic increase above 75% of GC content was present from 42,800 to 47,200 bp, in the region where tandem minisatellites were identified (Supplementary Fig. S4b). Two ORFs coding for hypothetical proteins and one ORF with high similarity to thrombospondin motifs were annotated in this region (Fig. 3a; Supplementary Table S10). A sharp drop to below 25% of GC content was observed from 47,300 to 49,800 bp, corresponding to the AT-rich stretch of tandem microsatellite repeats (Supplementary Fig. S4b). This partially palindromic 2.5 kb sequence was annotated as a *En/Spm* transposon-like element (Fig. 3a; Supplementary Table S11). A second increase of GC content (>75%) occurred from 49,900 to 52,150 bp, in the region with tandem minisatellites (Supplementary Fig. S4b), and an ORF with high similarity to a proline-rich protein (Fig. 3a; Supplementary Table S10). The GC content varied between 75% and 50% from 52,200 to 55,000 bp, where minisatellites were detected on the end of repeat-rich region (Supplementary Fig. S4b). Distinct switches in GC composition are used as genomic signatures of possible horizontal or lateral gene transfers particularly in bacteria^53,54^. Whether the observed fluctuations in GC content in the *P. brassicae*-specific mitochondrial region 42,800 to 55,000 bp region could be a remnant of ancient genomic events remain to be elucidated.

**Figure 5:**
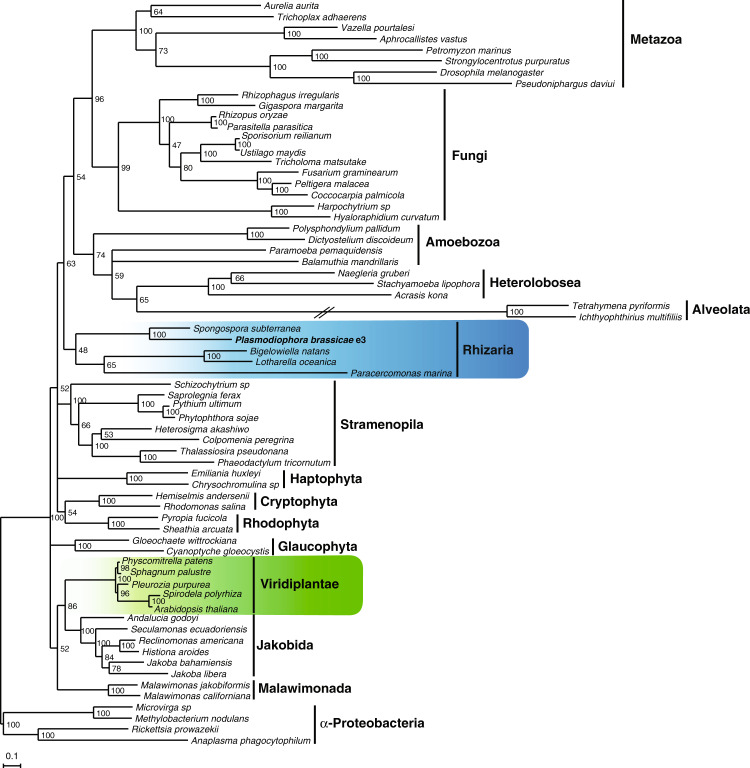
Maximum likelihood tree inferred from mitochondrial-encoded proteins. The tree was constructed from a concatenated alignment of 12 mitochondrial protein sequences from 67 organisms (63 from major eukaryotic groups and 4 α-protebacteria used as outgroup species). The super-matrix was analyzed with RAxML 8.2.11^60^ using GAMMA and substitution models specified for each partition. For details, see Supplementary Table S15. Rapid bootstrap analysis was done with 250 iterations. Branches with support values <45 were collapsed. The scale bar shows the inferred number of amino acid substitutions per site. The long branch was reduced to 50% of its original length, indicated by a crossed double line.

The SAR group (Stramenopila, Alveolata, Rhizaria) comprises numerous diverse eukaryotic organisms on which revised phylogenetic relationships have been presented^2^. We attempted to generate new information based on the *P. brassicae* e3 mt genome, since no phylogeny is yet proposed for the Phytomyxea plant pathogens based on a larger set of mitochondrial genes. Amino acid sequences for 12 protein-coding genes (*cob*, *cox1*, *cox2*, *cox3*, *atp6*, *nad1*, *nad2*, *nad3*, *nad4*, *nad4L, nad5* and *nad6*) from 67 organisms were used to infer maximum likelihood RAxML single-gene and concatenated trees. Twelve single-gene trees showed a well-supported close relationship between *P. brassicae* and *S. subterranea* as well as between *B. natans* and *L. oceanica*. When 773 mt proteins were analyzed, the five species in Rhizaria clustered together and *P. brassicae* and *S. subterranea* remained the two most closely related plasmodiophorids in the concatenated tree (Fig. 5). Moderate support for Rhizaria and several other major clades most likely reflects limitations in sequence information. However, no evidence for horizontal gene transfer events to *P. brassicae* neither from any species in Viridiplantae including *Arabidopsis thaliana*, which can act as a host, nor from other organisms was detected.

## Conclusion

Our long-read sequencing approach provided new insights into the nuclear and mitochondrial genomes of the clubroot pathogen *P. brassicae*. For the first time telomeric sequences are presented in the supergroup Rhizaria. Whether *P. brassicae* and algae share evolutionary history is intriguing. Both the telomeric T_4_AG_3_ repeats and some mitochondrial ORFs in *P. brassicae* showed high sequence similarities with algae and point to that direction. The close relationship between *P. brassicae* and *S*. *subterranea* was further visualized upon mt genome comparisons. A number of peculiarities were found in the *P. brassicae* mt genome such as: a unique repeat-rich region, a new splicing pattern with the GT-AT and GA-CA acceptor-donor sites, group II introns spanning exon-intron boundaries, and sequence similarities in ORFs to functional genes present in various organisms.

## Methods

### Materials and sequencing

Resting spores from clubs of *Brassica rapa* grown in *Plasmodiophora brassicae* strain e3 infested soils were isolated and used for DNA extraction^55^. High-quality DNA was sent to SciLifeLab, Uppsala, Sweden for PacBio RSII sequencing according to the manufacturer’s protocol.

### Nuclear assembly and annotation

Raw reads were assembled using FALCON v0.4 and HAGP3 from SMRTportal v2.3. The two assemblies were manually merged and polished using Quiver from SMRTportal v2.3. The gene annotation pipeline MAKER v2.3^32^ was used in combination with *ab-initio* gene predictors: Augustus v2.5.5, SNAP and GeneMark-ES v2.3. Augusts and SNAP were trained on the previously annotated and manually curated *P. brassicae* nuclear genes^16^. The UniProt/Swiss-Prot database and all rhizarian ESTs and proteins found at NCBI, as well as transcripts assembled from strand-specific RNASeq *P. brassicae* libraries^16^ were used as evidence integrated in gene predictions. Annotation of repeats was performed within MAKER^32^, using a *P. brassicae* specific repeat library constructed *de novo* using RepeatModeler v1.0.7 as well as MAKER’s internal library of transposable elements and the Repbase repeat library rm-20130422. MAKER was run using default parameters except *pred_flank* that was set to 100 bp, *split_hit*. Telomeric sequences were identified by using Tandem Repeats Finder^24^. For biological pathway classifications we used the WebMGA and the KOG classification tools. Additional protein analysis was done using OrthoMCL, sequence similarity searches, BLASTP searches against GenBank non-redundant protein database, HMM-searches against Pfam database, and RPS-BLAST searches against NCBI KOG (March 2017).

### Mitochondrial genome *de novo* assembly and annotation

The contig encoding the mitochondrial genome was assembled using Canu v1.5^56^. Visualization of the raw assembly with Bandage generated a circular contig (133,222 bp) in which overlapping sequences were identified by Gepard v1.40^57^. After removing overlaps (18,559 bp) and circularization, the final 114,663 bp long PacBio sequence was polished using Quiver. Mapping of the Illumina data^16^ to the PacBio sequence using BWA v0.7.15 and SAMtools v1.5 and polishing with Pilon revealed 2 × 1 bp difference, which were corrected. Next, the coverage was tested by aligning the PacBio reads to the assembled mitochondrial genome using GraphMap v0.5.2^58^.

To optimize *de novo* annotation several tools and sources were used and combined with manual curation. Automated annotations were done using MFannot v1.33^41^ with the genetic code 4 “Mold, protozoan and coelenterate mitochondrial; Mycoplasma/Spiroplasma”, Prokka v1.1^42^ with mitochondria and archaea kingdoms and MAKER2^43^ using Repeatmasker^44^, tRNAscan-SE^45^, Uniprot/Swiss-Prot mitochondrial proteins (Nov. 2016), the ribosomal database^46^ and Rfam v12^47^. Further information was provided by transcriptome data^16^, re-assembled using Trimmomatic, Tophat and Stringtie. The *S. subterranea* annotated mitochondrial genome^48^ was used as an additional source. Based on the different lines of annotation and sources, the gene models have been manually created through Web Apollo^49^. Translated CDS features were blasted against the Uniprot/Swiss-Prot reference data set (Aug. 2016) and filtered using a maximal e-value of 10e-6 and run against InterProScan v5.21–60. All retrieved functional information have been integrated into the final annotated data set. Predicted ORFs were used as query sequences for sequence similarity searches of the Uniprot/Swiss-Prot database (March 2019).

### Mitochondrial sequence comparison

Dot-plots created by Gepard v1.40^57^ were used for comparison of *P. brassicae* mitochondrial sequences from four strains: e3 (generated in this study, GeneBank accession LS992577), eH^23^ (GeneBank accession POCA01000043), ZJ-1^22^ (GeneBank accession MCBL01000050) and the Pb3 strain^21^ (GeneBank accession RZOB01000060). Additional sequence comparison was done using two mitochondrial contigs of the e3 strain generated by Illumina/454, excluded from^16^ but provided in Supplementary Table S6. Synteny between mitochondrial sequences from four *P. brassicae* strains and *S. subterranea*^48^ (GeneBank accession KF738139) was tested using the Mauve genome alignment tool v2.4.0^38^ with default settings.

### Phylogenetic analysis

Amino acid sequences were retrieved from public databases for 12 mitochondrial protein-coding genes (*cob*, *cox1*, *cox2*, *cox3*, *atp6*, *nad1*, *nad2*, *nad3*, *nad4*, *nad4L, nad5* and *nad6*) conserved across 67 organisms. Sixty-three organisms were selected to represent major eukaryotic groups with available complete mitochondrial genomes and if possible, deep-branching positions. Four α-protebacteria were selected as outgroup species. The sequences were aligned using Clustal Omega v1.2.1^59^ with default settings. Multiple alignments were visualized with AliView, examined and automatically trimmed with trimAL v1.4. Maximum likelihood (ML) phylogenetic trees with rapid bootstrap (RB) analyses were generated with RAxML v8.2.11^60^. The best amino acid substitution model was estimated with PROTGAMMAUTO option for each single gene tree and run with 250 to 650 RB, a number of iterations predicted to be sufficient by the autoFC stopping criteria. For concatenated trees, 12 protein alignments were concatenated (the script is available at https://github.com/nylander/catfasta2phyml) into a super-matrix comprising 773 genes and 3,819 aligned amino acid positions. ML analyses were inferred under GAMMA rate of heterogeneity with the substitution models specified for each partition and run with 250 RB iterations. Trees were displayed and edited with Dendroscope v3.5.9^61^. Information on organisms and proteins and substitution models are listed in Supplementary Table S15.

## Supplementary information

Supplementary Information

Supplementary Table S3

Supplementary Table S6

Supplementary Table S15

## Data Availability

Data retrieved in this study are deposited in the European Nucleotide Archive under the project PRJEB24736. For the nuclear sequence under accession number OVEO01000001-OVEO01000020 and the mt sequence under accession number LS992577.

## References

[1] Foissner, W. & Hawksworth, E. *Protist diversity and geographical distribution* (eds Foissner, W & Hawksworth D). Vol. 8. Topics in biodiversity and conservation. (Springer, 2009).

[2] Cavalier-Smith T, Chao EE, Lewis R (2018). Multigene phylogeny and cell evolution of chromist infrakingdom Rhizaria: contrasting cell organization of sister phyla Cercocoa and Retaria. Protoplasma.

[3] Neuhauser S, Kirchmair M, Gleason FH (2011). Ecological roles of the parasitic phytomyxids (plasmodiophorids) in marine ecosystems: a review. Marine & Freshwater Res..

[4] Dixon GR (2009). The occurrence and economic impact of *Plasmodiophora brassicae* and clubroot disease. J. Plant Growth Regul..

[5] Wallenhammar A-C (2014). Clubroot, a persistent threat in Swedish oilseed rape production. Can. J. Plant Pathol..

[6] Botero A (2019). Clubroot disease in Latin America: distribution and management strategies. Plant Pathol..

[7] Ingram DS, Tommerup IC (1972). The life history of *Plasmodiophora brassicae* Woron. Proc. R. Soc. Lond. B..

[8] McDonald MR (2014). The role of primary and secondary infection in host response to *Plasmodiophora brassicae*. Phytopathol..

[9] Kageyma K, Asano T (2009). Life cycle of *Plasmodiophora brassicae*. J. Plant Growth Regul..

[10] Siemens J, Bulman S, Rehn F, Sundelin T (2009). Molecular biology of *Plasmodiophora brassicae*. J. Plant Growth Regul..

[11] Schwelm A, Dixelius C, Ludwig-Müller J (2016). New kid on the block – the clubroot pathogen genome moves the plasmodiophorids into the genomic era. Eur. J. Plant Pathol..

[12] Strelkov SE (2018). Virulence and pathotype classification of *Plasmodiophora brassicae* populations collected from clubroot resistant canola (*Brassica napus*) in Canada. Can. J. Plant Pathol..

[13] Curtis BA (2012). Algal genomes reveal evolutionary mosaicism and the fate of nucleomorphs. Nature.

[14] Glöckner G (2014). The genome of the foraminiferan Reticulomyxa filosa. Curr. Biol..

[15] Keeling PJ (2014). The marine microbial eukaryote transcriptome sequencing project (MMETSP): illuminating the function a diversity of eukaryotic life in the oceans through transcriptome sequencing. PLoS Biol..

[16] Schwelm A (2015). The *Plasmodiophora brassicae* genome reveals insights in its life cycle and ancestry of chitin synthases. Sci. Rep..

[17] Krabberød AK (2017). Single cell transcriptomics, mega-phylogeny, and the genetic basis of morphological innovations in Rhizaria. Mol. Biol. & Evol..

[18] Ciaghi S, Neuhauser S, Schwelm A (2018). Draft genome resource for the potato powdery scab pathogen *Spongospora subterranea*. Mol. Plant-Microbe Interact..

[19] Falloon RE (2016). Root infection of potato by *Spongospora subterranea*: knowledge review and evidence for decreased plant productivity. Plant Pathol..

[20] Sierra R (2016). Evolutionary origins of Rhizarian parasites. Mol. Biol. Evol..

[21] Rolfe SA (2016). The compact genome of the plant pathogen *Plasmodiophora brassicae* is adapted to intracellular interactions with host *Brassica* spp. BMC Genomics.

[22] Bi K (2016). Integrated omics study of lipid droplets from *Plasmodiophora brassicae*. Scientific Rep..

[23] Daval, S. *et al*. Computational analysis of the *Plasmodiophora brassicae* genome: mitochondrial sequence description and metabolic pathway database design. *Genomics*10.1016/j.ygeno.2018.11.013 10.1016/j.ygeno.2018.11.013 30447277

[24] Benson G (1999). Tandem repeats finder: a program to analyze DNA sequences. Nucleic Acids Res..

[25] Petracek ME, Lefebvre PA, Silflow CD, Berman J (1990). Chlamydomonas telomere sequences are A+T-rich but contain three consecutive G-C base pairs. Proc. Nat. Acad. Sci. USA.

[26] Sohanpal BK, Morzaria SP, Gobright EI, Bishop RP (1995). Characterisation of the telomeres at opposite ends of a 3 Mb *Theileria parva* chromosome. Nucleic Acids Res..

[27] Fredrychova RC, Mason JM (2013). Telomeres: their structure and maintenance. In: Stuart D., ed. Mechanisms of DNA replication. INTECH Open Science.

[28] Procházková Schrumpfová P, Schořová Š, Fajkus J (2016). Telomere- and telomerase-associated proteins and their functions in the plant cell. Front. Plant Sci..

[29] Mehrabi R, Gohari AM, Kema GHJ (2017). Karyotype variability in plant-pathogenic fungi. Ann. Rev. Phytopathol..

[30] Croll D, McDonald BA (2012). The accessory genome as a cradle for adaptive evolution in pathogens. PLoS Pathog..

[31] Strehlow B, de Mol F, Struck C (2014). History of oilseed rape cropping and geographic origin affect the genetic structure of *Plasmodiophora brassicae* populations. Phytopathol..

[32] Cantarel BL (2008). MAKER: An easy-to-use annotation pipeline designed for emerging model organism genomes. Genome Res..

[33] Braselton JP (1982). Karyotypic analysis of *Plasmodiophora brassicae* based on serial thin-sections of pachytene nuclei. Can. J. Bot..

[34] Giroux CN, Dresser ME, Tiano HF (1989). Genetic control of chromosome synapsis in yeast meiosis. Genome.

[35] Qiao H (2012). Interplay between synaptonemal complex, homologous recombination, and centromeres during mammalian meiosis. PLoS Genet..

[36] Grishaeva TM, Bogdanov YF (2014). Conservation and variability of synaptonemal complex proteins in phylogenesis of eukaryotes. Int. J. Evol. Biol..

[37] Gao J, Colaiácova MP (2018). Zipping and unzipping: protein modifications regulating synaptonemal complex dynamics. Trends Genet.

[38] Darling ACE, Mau B, Blattner FR, Perna NT (2004). Mauve: Multiple alignment of conserved genomic sequence with rearrangements. Genome Res..

[39] Oren M (2016). Short tandem repeats, segmental duplications, gene deletion, and genomic instability in a rapidly diversified immune gene family. BMC Genom..

[40] Paajanen P (2019). A critical comparison of technologies for a plant genome sequencing project. Gigascience.

[41] Beck, N. & Lang, B.F. MFannot, organelle genome annotation webserver. http://megasun.bch.umontreal.ca/cgi-bin/mfannot/mfannotInterface.pl (2010).

[42] Seemann T (2014). Prokka: rapid prokaryotic genome annotation. Bioinformatics.

[43] Holt C, Yandell M (2011). MAKER2: an annotation pipeline and genome-database management tool for second-generation genome projects. BMC Bioinformatics.

[44] Smit, A.F.A., Hubley, R. & Green, P. RepeatMasker Open-3.0. http://www.repeatmasker.org (2010).

[45] Schattner P, Brooks AN, Lowe TM (2005). The tRNAscan-SE, snoScan and snoGPS web servers for the detection of tRNAs and snoRNAs. Nucleic Acids Res..

[46] Cole JR (2014). Ribosomal Database Project: Data and tools for high throughput rRNA analysis. Nucleic Acids Res..

[47] Nawrocki EP (2015). Rfam 12.0: updates to the RNA families database. Nucleic Acids Res..

[48] Gutiérrez P, Bulman S, Alzate J, Ortíz MC, Marin M (2016). Mitochondrial genome sequence of the potato powdery scab pathogen *Spongospora subterranea*. Mitochondrial DNA Part A.

[49] Lee E (2013). Web Apollo: a web-based genomic annotation editing platform. Genome Biol..

[50] Tanifuji G, Archibald JM, Hashimoto T (2016). Comparative genomics of mitochondria in chlorarachniophyte algae: endosymbiotic gene transfer and organellar genome dynamics. Sci. Rep..

[51] Valach M, Moreira S, Kiethega GN, Burger G (2014). Trans-splicing and RNA editing of LSU rRNA in *Diplonema* mitochondria. Nucleic Acids Res..

[52] Guha TK, Wai A, Mullineux S-T, Hausner G (2018). The intron landscape of the mtDNA *cytb* gene among the Ascomycota: introns and intron-encoded open reading frames. Mitochondrial DNA Part A.

[53] Hayek N (2013). Lateral transfer and GC content of bacterial resistance genes. Front. Microbiol..

[54] Zhang D (2014). Root parasitic plant *Orobanche aegyptiaca* and shoot parasitic plant *Cuscuta australis* obtained Brassicaceae-specific strictosidine synthase-like genes by horizontal gene transfer. BMC Plant Biol..

[55] Mehrabi S, Stjelja S, Dixelius C (2018). Disease establishment, resting spore isolation and DNA extraction of *Plasmodiophora brassicae*, the clubroot pathogen. Bio-Protocol.

[56] Koren S (2017). Canu: scalable and accurate long-read assembly via adaptive *k*-mer weighting and repeat separation. Genome Res..

[57] Krumsiek J, Arnold R, Rattei T (2007). Gepard: a rapid and sensitive tool for creating dotplots on genome scale. Bioinformatics.

[58] Sović I (2016). Fast and sensitive mapping of nanopore sequencing reads with GraphMap. Nat. Com..

[59] Sievers F (2011). Fast, scalable generation of high-quality protein multiple sequence alignments using Clustal Omega. Mol. Systems Biol..

[60] Stamatakis A (2014). RAxML version 8: A tool for phylogenetic analysis and post-analysis of large phylogenies. Bioinformatics.

[61] Huson D, Scornavacca C (2012). Dendroscope 3: An interactive tool for rooted phylogenetic trees and networks. System. Biol..

