# Author Correction: The architecture of the *Plasmodiophora brassicae* nuclear and mitochondrial genomes

**DOI:** 10.1038/s41598-022-09753-1

**Published:** 2022-04-06

**Authors:** Suzana Stjelja, Johan fogelqvist, Christian Tellgren-Roth, Christina Dixelius

**Affiliations:** 1 Department of Plant Biology, Uppsala BioCenter, Linnéan Center for Plant Biology, Swedish University of Agricultural Sciences, P.O. Box 7080, SE-75007 Uppsala, Sweden; 2 Uppsala Genome Center, Science for Life Laboratory, Department of Immunology, Genetics and Pathology, Uppsala University, BMC, Box 815, SE-751 08 Uppsala, Sweden

Correction to: *Scientific Reports* https://doi.org/10.1038/s41598-019-52274-7, published online 31 October 2019

The Supplementary Information file published with this Article contained an error in Table S4 where a previous rendition was published. The original Supplementary Information file is provided below.

This error has now been corrected in the Supplementary Information file that accompanies the original Article.

## Supplementary Information

Supplementary Information.

