# Supplementary material for: The architecture of the Plasmodiophora brassicae nuclear and mitochondrial genomes
Source: Sci Rep. 2019 Oct 31;9:15753. doi: 10.1038/s41598-019-52274-7 (PMC6823432; doi:10.1038/s41598-019-52274-7)
Supplement: Supplementary file 1 — Supplementary Information [file 41598_2019_52274_MOESM1_ESM.docx]

**Supplementary Information**

**The architecture of the *Plasmodiophora brassicae* nuclear and mitochondrial genomes**

**Suzana Stjelja, Johan Fogelqvist, Christian Tellgren-Roth and Christina Dixelius**

**Supplementary Figures S1-S9**

**Supplementary Tables S1-S15**

**Supplementary Notes**

**Supplementary References**

**Supplementary Figures**

contig_01_5' 1 -------------------------------TAGGGTTTT------------TTAGGGTT

contig_02_5' 1 -------------------ATTATTAGGGTTTTTTAGGG---TTTTTTAGGGTTT-----

contig_03_5' 1 -GTTTTTAGGGT-----AGGGTTTTAGGGTTTTTTAGGG---GCTT--AGGGTTTT-AGG

contig_04_5' 1 GGGTT-------TTTTAGGGTTTTTTAG--------TTTAGGGTT--------------T

contig_05_5' 1 -------AGGGTTTTTTAGGGGCTTAGGGTTTT--AGGG---TTTT--AGGGGCTT-AGG

contig_06_5' 1 GGTTTTTAGGGTTTT--AGGGTTTTAGGGTTTT--AGGG---TTTT--AGGGTTTT-AGG

contig_07_5' 1 --CGGGTTTTTTAGGGTTTT-----------------TTAGGGTTTTTTAGGGGTTTTTT

contig_08_5' 1 -----------TTTT--AGGGTTTTAGGGTTTTTTAGGG---TTTT--TAGGTT---AGG

contig_09_5' 1 GGGTTTTAGGGTTTT--AGGGTTTTAGGGTTTT--AGGG---TTTT--AGGGTTTT-AGG

contig_11_5' 1 TTAGGGTTTTTTAGG-----GTTTTAGGGTT--------------TTTAGGGTTTTT---

contig_12_5' 1 ----------GTTT---AGGGTTTTAGGGTTTTTTAGGG---CTT---AGGGTTTT-AGG

contig_13_5' 1 ------------------------TAGGGTTTTTTAGGGGTTTTTTTTAGGGTTTTTAGG

contig_14_5' 1 ------TAGGGCTT---AGGG------GGTTTTTTAGGG---TTTTT-AGGGTTTTTAGG

contig_17_5' 1 AGGGGTT-------------TTTTAGGGTTTT---------TTTT--AGGGTTTTT---A

contig_18_5' 1 TCGGATTAGGGTTT--------TTTAGGGCTTTTTAGGG---TTTTTTAGGGTTTTTAGG

contig_19_5' 1 ----------------------------TTAGGGTTTTT----------TT--AGGGTTT

contig_20_5' 1 ----CTTAGGGTTTT--AGGGTTTTAGGGTTTT--AGGG---TTTT--AGGGTTTT-AGG

contig_01_3' 1 ---TTTTAGGGTTTT--AGGGTTTTAGGGTTTT--AGGG---TTTT--AGGGTTTTTAGG

contig_02_3' 1 AGGGCTTAGGGTTTT--AGGGTTTTAGGGTTTTTTAGGG---TTTTTTAGGGTTTT-AGG

contig_03_3' 1 -----TTAGGGTTTTTTTAGGGTTTAGGGTTTT--AGGG---TTTT--AGGGTTTT-AGG

contig_04_3' 1 ---TTTTAGGGTTTT--AGGGTTTTAGGGTTTTTTAGGG---TTTT--AGGGTTTT-AGG

contig_06_3' 1 -------AGGGTTTT--AGGGTTTTAGGGTTTT--AGGG---TTTT--AGGGTTTTAGGG

contig_08_3' 1 -GGTTTTAGGGTTTT----GGTTTTAGGGTTTT--AGGG---TTTT--AGGGTTTT-AGG

contig_09_3' 1 -----TTAGGGTTT---AGGGTTTTAGGGTTTTT-AGGG---TTTT--AGGGTTTT-AGG

contig_12_3' 1 ---TTTTAGGGTTTT--AGGGTTTTAGGGTTTT--AGGG---TTTT--AGGGTTTT-AGG

contig_13_3' 1 GGGTTTTAGGGTTTT--AGGGTTTTAGGGTTTT--AGGG---TTTT--AGGGTTTT-AGG

contig_14_3' 1 -----TTAGGGGTTT--AGGGGTTTAGGGTTTTTTAGGG---TTTT--AGGGTTTT-AGG

contig_16_3' 1 ------TAGGGTTTT--AGGGTTTTAGGGTTTT--AGGG---TTTTT-AGGGTTTT-AGG

contig_17_3' 1 GAGCGGT----GCCTGGGGGCTTTAGGGTTTTA----------------GGTTT-----A

contig_18_3' 1 GTCGGGTTTT--------TTTAGGGTTTTTTAGGC-------------------------

contig_20_3' 1 GGTTTTTAGGGTTTT--AGGGCTTAGGGTTTTT--AGGG---TTTT--AGGGTTTTTAGG

contig_01_5' 18 TTTAGGGTTTT-------------------------------------------------

contig_02_5' 34 --TTTTAGGGTTTT--AGGGTTT---AGGGTTTTTAGGGTTTT------TTTTAGGGTTT

contig_03_5' 49 G--TTTAGGGTTTT--AGGGTTTT--AGGGTTTT-TGGGCTT--AGGGTTTTTAGGGTTT

contig_04_5' 32 TTTAGGTTTTA--TTAGGGTTTTTTAGGGTTTT----AGGGGCTTTTTAGGTTAGGGGGT

contig_05_5' 46 G-TTTTAGGGTTTT--AGGGGGTT--AGGGTTTT-AGGGTTTT-AGGG-TTTTAGGGTTT

contig_06_5' 51 G-TTTTAGGGTTTT--AGGGTTTT--AGGGTTTT-AGGGTTTT-AGGG-TTTTAGGGTTT

contig_07_5' 42 TTAGGGTT--------------TTTAGGGTTTTT--------TTTTAGGGGGTTTT----

contig_08_5' 40 G-TTTTAGGGTTTTT-AGGGTTT---AGGGTTTT-AGGGTTTT-AGGG-TTTTAGGGTTT

contig_09_5' 51 G-TTTTAGGGTTTT--AGGGTTTT--AGGGTTTT-AGGGCTT--AGGGTTTTTAGGGTTT

contig_11_5' 39 -----TTTT--AGGGTTTT----AGGGTTTTTTAGGGTTTTAGGGTTTT-----------

contig_12_5' 41 G-TTTTAGGGTTTT--AGGGTTTTTTAAGGGCTT-AGGGTTTT-AGGG-TTTTAGGGTTT

contig_13_5' 37 G-TTTTAGGGTTT---AGGGGGTTTTAGGGTTTT-AGGGTTTTAGGGTTTTTTAGGGTTT

contig_14_5' 42 C---TTAGGGTTTTTAAGGGTTTTTTAGGGTTTTTAGGGTTTT-AGGG-TTTTAGGGTTT

contig_17_5' 34 GGGTTTTTTAGGGTT-----------------------------TTTTAGGCTT------

contig_18_5' 50 G-TTTTAGGGCTT---AGGGTTTTTTAGGGGCTT-AGGGTTT--------TTTAGGGTTT

contig_19_5' 21 T-------------------TTTAGGGTTTT--------------------TTTAGGGTT

contig_20_5' 47 G--TTTAGGGTTTT--AGGGTTT---AGGGCTT--AGGGTTTT-AGGGTTTTTAGGGTTT

contig_01_3' 49 G-TTTTAGGGTTTT--AGGGTTTT-------TTT-AGGGTTTT-AGGG-TTTTAGGGTTT

contig_02_3' 55 G-TTTTAGGGTTTT--AGGGTTTT--AGGGTTTT-AGGGTTTTAGGG--TTTTAGGGTTT

contig_03_3' 48 G-TTTTAGGGTTTT--AGGGTTTT--AGGGTTTT-AGGGTTTTAGGG--TTTTAGGGTTT

contig_04_3' 50 G-TTTTAGGGTTTT--AGGGTTTT--AGGGTTTT-AGGGTTTT-AGGG-TTTTAGGGTTT

contig_06_3' 45 GTTTTTAGGGTTTT--AGGGTTTTT-AGGGTTTT-AGGGTTTTTAGGGGTTTTAGGGTTT

contig_08_3' 48 G-TTTTAGGGTTTT--AGGGTTTT--AGGGTTTT-AGGGTTTTTAGGG-TTTTAGGGTTT

contig_09_3' 46 GTTTTTAGGGTTTT--AGGGTTTT--AGGGTTTT-AGGGTTTT-AGGG-TTTTAGGGTTT

contig_12_3' 48 G-TTTTAGGGTTTT--AGGGTTT----AGGTTTT-AGGGTTTT-AGGG-TTTTAGGGTTT

contig_13_3' 51 G-TTTTAGGGTTTT--AGGGTTTT--AGGGTTTT-AGGGTTT--AGGGTTTTTAGGGTTT

contig_14_3' 48 G-TTTTAGGGTTTT--AGGGTTTTTTAGGGTTTT-AGGGTTTT-AGGG-TTTTAGGGTTT

contig_16_3' 46 G-TTTTAGGGTTTT--AGGGTTTTT-AGGGTTTT-AGGGTTTT-AGGG-TTTTAGGGTTT

contig_17_3' 36 GGGTTTTTTAGGGTTTTAGGGTTAGGGGGTT-------------TTTT------------

contig_18_3' 28 TTAGGGTTTT--AGGGTTTT--AGGGTTTT-AGGGTTA----GGGTTTTAGGGTTTT---

contig_20_3' 52 G---TTAGGGTTTT--AGGGTTTT--AGGGTTTT-AGGGTTTT-AGGG-TTTTAGGGTTT

contig_01_5' ------------------------------------------------------------

contig_02_5' 81 T-----------TTTTAGGTTTTTAGGGTTTAGGGTTTTAGGGTTTTA-----GGGTTTT

contig_03_5' 100 T-TAGG-----GTTTTAGGGTT-------TTAGGGTTTTAGGGTTTTA-----GGGTTTT

contig_04_5' 86 TTTTT-------------------------------------------------------

contig_05_5' 98 T--AGG-----GTTTTAGGGTT-------TTAGGGTTTTAGGGTTTTA-----GGGTTTT

contig_06_5' 103 T--AGG-----GTTTTAGGGTT-------TTAGGGTTTTAGGGTTTTA-----GGGTTTT

contig_07_5' 76 -------------TTTTTAGGGTTTT-------------TAGG-GTTTTAGGGTTTT---

contig_08_5' 92 T--AGG-----GTTTTAGGGTT-------TTAGGGTTTTAGG-TTTTA-----GGGTTTT

contig_09_5' 103 T-----------TTTTAGGGTT-------TTAGGGTTTTAGG--TTTA-----GGGTTTT

contig_11_5' 77 --------TTTTAGGG--------------------------------------------

contig_12_5' 95 T--AGG------TTTTAGGGTT-------TTAGGGTTTTAGGGTTTTT----AGGGTTTT

contig_13_5' 92 TTTAGG-----GCTTAGGGGCT-------TTAGGGTTTTAGGGTTTTAGTTTTAGGTTTT

contig_14_5' 97 T--AGG-----GTTTTAGGGTT-------TTAGGGTTTTAGGGTTTTA-----GGGTTTT

contig_17_5' ------------------------------------------------------------

contig_18_5' 97 T-----------TTTTAGGGTT-------TTAGGGTTTTAGGGTTTTA-----GGGTTTT

contig_19_5' 42 TTT---------------------------------------------------------

contig_20_5' 97 T--AGG-----GTTTTAGGGTT-------TTAGGGTTTTAGGGTTTTA-----GGGTTTT

contig_01_3' 96 T--AGG-----GTTTTAGGGTT-------TTAGGGTTTTAGGGTTTTA-----GGGTTT-

contig_02_3' 107 T--AGG------TTTTAGGTTT---------AGGGTTTTAGGGTTTTA-----GGGTTTT

contig_03_3' 100 T--AGG-----GTTTTAGGGTTGGGTTTTTTAGGGTTTTAGGGTTTTA-----GGGTTTT

contig_04_3' 102 T--AGG-----GTTTTAGG-TT-------TTAGGGTTTTAGGGTTTTA-----GGGTTTT

contig_06_3' 101 T--AGG-----GTTTTAGGGTT-------TTAGGGTTTTAGGGTTTTA-----GGGTTTT

contig_08_3' 101 T--AGG-----GTTTTAGGGTT-------TTAGGGTTTTAGGGTTTTA-----GGGTTTT

contig_09_3' 99 T--AGG-----GTTTTAGGGTT-------TTAGGGTTTTAGGGTTTTA-----GGGTTTT

contig_12_3' 98 T--AGG-----GTTTTAGGGTT-------TTAGGGTTTTAGGGTTTTATTTTAGGGTTTT

contig_13_3' 103 T--AGG-----GTTTTAGGGTT-------TTAG--TTTTAGGGTTTTA-----GGGTTTT

contig_14_3' 102 T--AGG-----GTTTTAGGGTT-------TTAGGGTTT-AGGGTTTTA-----GGGTTTT

contig_16_3' 99 T--AGG-----GTTTTAGGGTT-------TTAGGGTTTTAGGGTTTTA-----GGGTTTT

contig_17_3' ------------------------------------------------------------

contig_18_3' 76 -----------TAGGGTT-------TTAGGGTTTTAGGGTTTTA-----GGGTTTTTTAG

contig_20_3' 102 T--AGG-----GTTTTAGGGTT-------TTAGGGTTTTAGGGTTTTA-----GGGTTTT

contig_01_5' ------------------------------------------------------------

contig_02_5' 125 TTAGGGTTTT----AGGGTTTTTTAGGGTTTTAGCGCGTTGGG----------TAGGGCT

contig_03_5' 142 --AGGGTTTTT---AGGGTTTT--AGGGTTTTAGGGTTTTAGGGTTT-----TTAGG-GT

contig_04_5' ------------------------------------------------------------

contig_05_5' 139 --AGGGTTTT----AGGGTTTT------TTTTAGGGTTTTAGGGTTTAGGGTTTAGG-GT

contig_06_5' 144 --AGGGTTTT----AGGGTTTT--AGGGTTTTAGGGTTTTAGGGTTT------TAGG--T

contig_07_5' ------------------------------------------------------------

contig_08_5' 132 --AGGGTTTT----AGGGTTTT--AGGGTTTTAGGGTTTTAGGGTTT-----TTAGG-GT

contig_09_5' 138 --AGGGTTTTT---AGGGTTT---AGGGTTTTAGGGTTT-AGGGTTT------TAGG-GT

contig_11_5' ------------------------------------------------------------

contig_12_5' 136 --AGGGTTTT----AGGGTTTT--AGGGTTTTAGGGTTTTAGGGTTT------TAGG-GT

contig_13_5' 140 --AGGGTTTT----AGGGTTTT--AGGGTTTTAGGGTTTTAGGGTTT------TAGG-GT

contig_14_5' 138 --AGGGTTTT----AGGGTTTT--AGGGTTTTAGGGTTTTAGGGTTT------TAGG-GT

contig_17_5' ------------------------------------------------------------

contig_18_5' 134 --AGGGTTTT----AGGGTTTT--AGGGTTTTAGGGTTTTAGG---------------GT

contig_19_5' ------------------------------------------------------------

contig_20_5' 138 --AGGGTTTT----AGGGTTTT--AGGGTTTTAGGGTTTTAGGGTTT------TAGG-GT

contig_01_3' 136 --AGGGTTTTTTTTAGGGTTTT-TAGGGTTTTAGGGTTTT-----TT------TAGG-GT

contig_02_3' 145 --AGGGTTTT----AGGGTTTTT-AGGGTTTTAGGGTTTTAGGGTTT------TAGGGGT

contig_03_3' 148 TTAGGGTTTT----AGGGTTT------TTTTTAGGGTTTTAGGGTTT-----TTAGG-GT

contig_04_3' 142 --AGGGTTTT----AGGGTTTT--AGGGTTTTAGGGTTTTAGGGTTT------TAGG-GT

contig_06_3' 142 --AGGGTTTTTTTTAGGGTTTT--AGGGTTTTAGGGTTTTAGGGTTT------TAGG-GT

contig_08_3' 142 --AGGGTTTT----AGGGTTTTAGAGGGTTTTAGGGTTTTAGG---T------TAGG-GT

contig_09_3' 140 --AGGGTTTT----AGGGTTTT-TAGGGTTTTAGGGTTTTAGGGTTT------TAGGGGT

contig_12_3' 144 --AGGGTTTTT---AGGGTTTT--AGGGTTTTAGGGTTTTAGGGTTT------TAGG-GT

contig_13_3' 142 TTAGGGGTTT----AGGGTTTT--AGGGTTTTAGGGTTTTAGGGTTT------TAGG-GT

contig_14_3' 142 --AGGGTTTT----AGGGTTTT--AGGGTTTTAGGGTTTTAGGGTTT------TAGG-GT

contig_16_3' 140 AGGGGGTTTT----AGGGTTTA----GGTTTTAGGGTTT-AGGGTTT------TAGG--G

contig_17_3' ------------------------------------------------------------

contig_18_3' 113 GGTTTT----AGGGTTTT-TAGGGTTTTAGGGTTTTAGGGTTT------TAGG-GTTTTA

contig_20_3' 143 --AGGGTTTT----AGGGTTTT--AGGGTTTTAGGGTTTT-------------TAGG-GT

contig_01_5' -----------------------

contig_02_5' 171 TTTA-------------------

contig_03_5' 189 TTTAGGGTTTTA-----------

contig_04_5' -----------------------

contig_05_5' 186 TTTAGGGTTTTAGGG--------

contig_06_5' 188 TTTAGGGTTTTAG----------

contig_07_5' -----------------------

contig_08_5' 178 TTTAGGGTTTTTAGGCGCTTTTT

contig_09_5' 182 TTTAGGGTTTTAGGGTTTA----

contig_11_5' -----------------------

contig_12_5' 181 TTTAGGGTTTTAGGGTTTTA---

contig_13_5' 185 TTTAGGGTTTTAGGGT-------

contig_14_5' 183 TTTAGGGTTTTAGGGTTT-----

contig_17_5' -----------------------

contig_18_5' 171 TTTAGGGTTTTAGG---------

contig_19_5' -----------------------

contig_20_5' 183 TTTAGGGTTT--AGGTTTTA---

contig_01_3' 181 TTTAGGGTTTTAGGTTTTAG---

contig_02_3' 192 TTTAGGGTT--------------

contig_03_3' 192 TTTAGGTTT--------------

contig_04_3' 187 TTTAGGGTTTTAGG---------

contig_06_3' 191 TTTAGGGTTT-------------

contig_08_3' 186 TTTAGGGTTTTAGGT--------

contig_09_3' 187 TTTAGGGTTTTAGG---------

contig_12_3' 190 TTTAGGGTTTT------------

contig_13_3' 189 TTTA---TTTTAGG---------

contig_14_3' 187 TTTAGGGTTTTAGG---------

contig_16_3' 183 TTTAGGGTTTTAGGGTTT-----

contig_17_3' -----------------------

contig_18_3' 161 GGGTTT-----------------

contig_20_3' 181 TTTAGGGTTTTAGGGTTTTA---

**Supplementary Fig. S1.** **Telomere sequences.** Alignments of *P. brassicae* e3

contig sequences showing consensus of telomere sequences in red.

**Supplementary Fig. S2. Protein families in Rhizaria and *P. brassicae*.** Functional annotation of *P. brassicae* e3 protein-encoding nuclear genes and comparisons with 12 other species in Rhizaria (Supplementary Table S3) based on OrthoMCL analyses^1^ and presented as KOG functional categories. The core is defined as presence of one protein-encoding gene in *P. brassicae*, *Spongospora subterranea*^2^ and in 8 or more species^3^.

**Supplementary Fig. S3.** **Synaptonemal complex-like genes in *P. brassicae* e3**. Expression patters (RLD, regularized log-transformed) of the synaptonemal complex-like genes in enriched life-stages (Ls) of *P. brassicae* e3^2^: Ls1 = germinating resting spores, Ls2 = mature resting spores, Ls3 = mixture of various life-stages including zoospores, Ls4= plasmodia. For details on genes, see Supplementary Table S4.


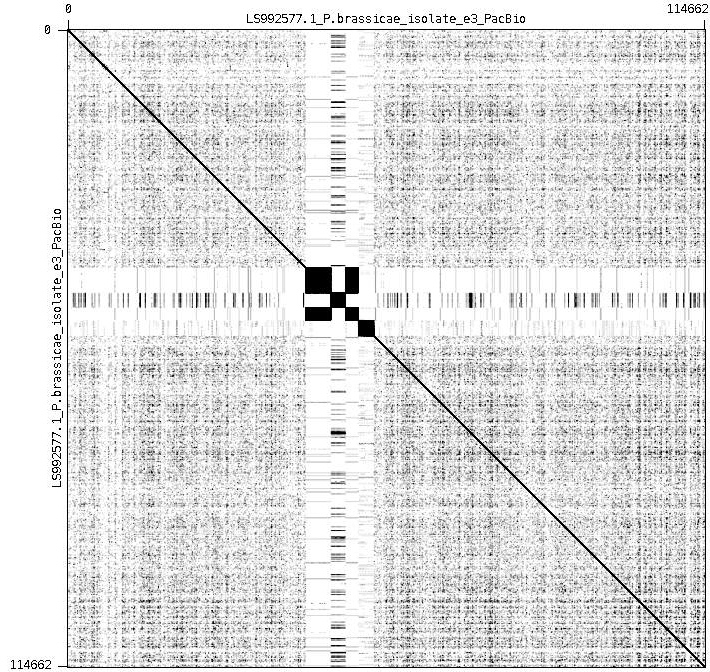


**Supplementary Fig. S4a. Self-similarity dot-plot of the *P. brassicae* e3 mitochondrial sequence.** The Gepard dot-plot^4^ with a continuous main diagonal illustrates perfect similarity. Black rectangles indicate a 12,500 bp long repeat-rich region spanning from 42,650 bp to 55,150 bp.

**Supplementary Fig. S4b.** **Close-up of the repeat-rich region in Supplementary Fig. S4a**. Block 1, 3 and 4 represent tandem minisatellite- and block 2 microsatellite repeats. Block 5 and 6 symbolize repeats with partially palindromic sequences.


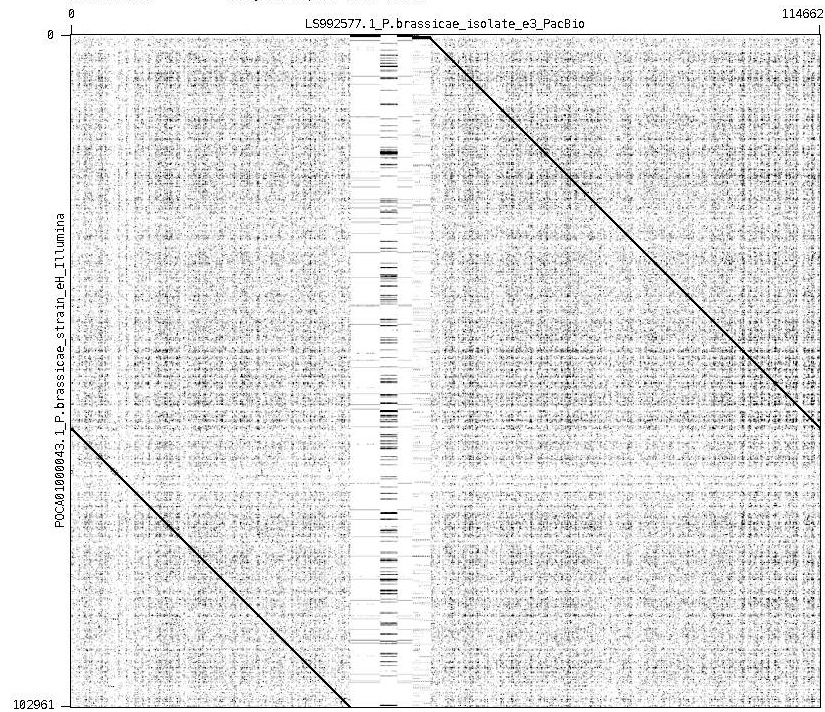


**Supplementary Fig. S5. Dot-plot of mitochondrial sequences.** Comparison between the *P. brassicae* e3 and eH**^5^** strains. The Gepard dot-plot^4^ illustrates a large discrepancy in the main diagonal from 42,600 to 55,000 bp. These positions correspond to the location of the repeat-rich region identified in the e3 sequence (Supplementary Fig. S4a,b).


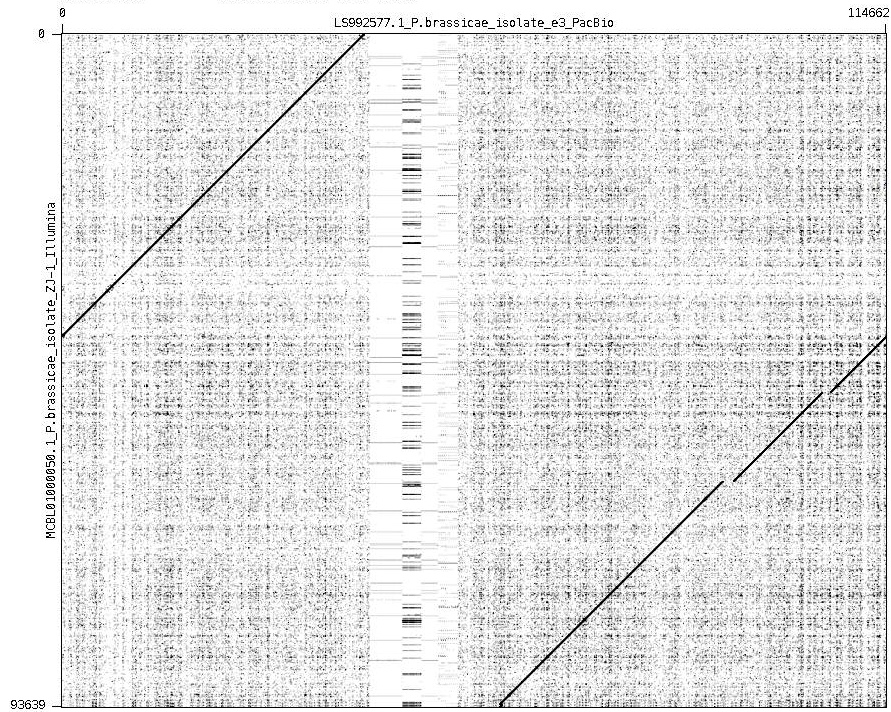


**Supplementary Fig. S6. Dot-plot of mitochondrial sequences.** Comparison between the *P. brassicae* e3 and ZJ-1^6^ strain. The Gepard dot-plot^4^ illustrates several discrepancies in the main diagonal, including a large region from 42,000 to 61,000 bp. These positions correspond to the location of the repeat-rich region identified in the e3 sequence (Supplementary Fig. S4a,b).


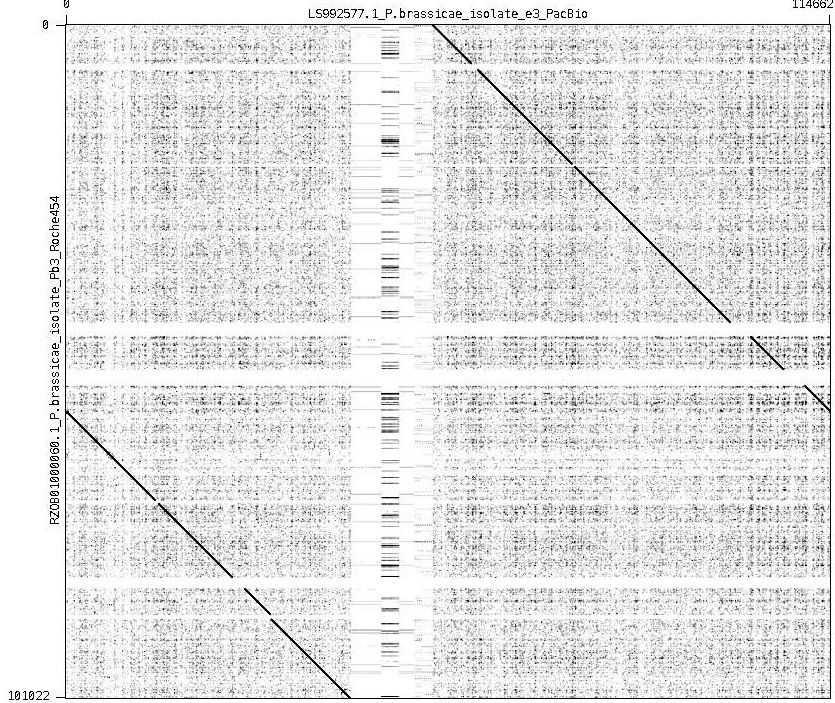


**Supplementary Fig. S7. Dot-plot of mitochondrial sequences.** Comparison between the *P. brassicae* e3 and Pb3^7^ strain. The Gepard dot-plot^4^ illustrates several discrepancies in the main diagonal, including a large region from 42,750 to 55,100 bp. These positions correspond to the location of the repeat-rich region identified in the e3 sequence (Supplementary Fig. S4a,b).


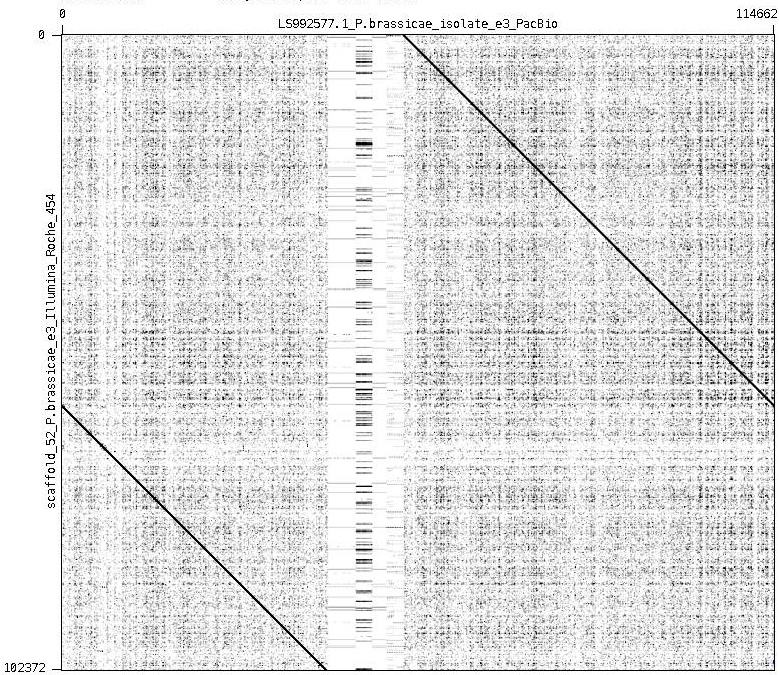


**Supplementary Fig. S8. Dot-plot of mitochondrial sequences.** Comparison between the PacBio and Illumina/454 mitochondrial sequences of *P. brassicae* e3. The Gepard dot-plot^4^ illustrates a large discrepancy in the main diagonal from 42,700 to 55,000 bp. These positions correspond to the location of the repeat-rich region identified in the PacBio e3 sequence (Supplementary Fig. S4a,b). The Illumina/454 e3 mitochondrial sequence information can be found in Supplementary Table S6.

**Supplementary Fig. S9. Synteny between four *P. brassicae* mitochondrial genomes.** A synteny image^8^ was adapted to facilitate comparisons. Identified homologous regions are displayed as two locally collinear blocks (LCB), green and red. The LCBs with matching colors between the genomes are connected by lines. Inside each LCB a sequence similarity profile is illustrated, with its height representing the average level of sequence conservation. White areas within a LCB indicate sequences that are unique to a particular genome and dark grey areas represent missing nucleotides. The region outside LCBs, here marked with a blue frame in the e3 strain, has no detectable homology in the other genomes.

**Supplementary Tables**

**Supplementary Table S1.** **Comparison between PacBioSII and Illumina/454 sequencing.** Statistics of *P. brassicae* e3 nuclear genome sequence data.

|  | PacBioRSII | Illumina/454^2^ |
| --- | --- | --- |
| Nuclear genome size (Mb) | 25 | 24 |
| No. assembled scaffolds | 20 | 165 |
| Scaffold N50 | 1.3 Mb | 473 kb |
| GC content % | 59 | 58 |
| Repeats and transposable elements % | 11.5 | 5.4 |
| No. predicted genes | 9,231 | 9,731 |

**Supplementary Table S2. Contig sizes of**

**the *P. brassicae* e3 nuclear and mitochondrial**

**genomes.**

| **Contig no.** | **Length (bp)** |
| --- | --- |
| 1  2  3  4  5  6  7  8  9  10  11  12  13  14  15  16  17  18  19  20  21 mt | 2120846  1933843  1875601  1681223  1483127  1452332  1368189  1360625  1287707  1285129  1270155  1249881  1090750  1031062  891373  867297  799070  759975  746515  692149  114663 |

**Supplementary Table S3.** ***P. brassicae* e3 protein coding genes present in 8 or more Rhizaria species based on OrthoMCL analysis.** AMAM *Amorphochlora amoebiformis*; AMSP *Ammonia sp*.; BILO, BINA, BINB, BINC, BNAT *Bigelowiella* sp.; CHRE *Chlorarachnion reptans*; ELMA *Elpidium margaritaceum;* GYSP *Gymnochlora* sp.; LOGA, LOGB, LOBC, *LOOC Lotharella* sp.; MICH *Minchinia chitonis*; NOSP *Norrisiella sphaerica*; PAGL *Partenskyella* *glossoppdia*; RFIL *Reticulomyxa filosa*; SPON *Spongospora subterranea* (Excel file).

**Supplementary Table S4**. **Orthologs of synaptonemal complex and meiosis-related genes in *P. brassicae* e3.** Regularized log transformed (RLD) expression levels in different enriched life-stages (Ls) are listed. Ls1 = geminating spores, Ls2 = mature spores, Ls3 = mixture of various life-stages including zoospores, Ls4= plasmodia, according to^2^.

| **Protein^a^** | **Org. ^b^** | **Accession no.** | ***P. brassicae* ID** | **Identity %** | **E-value** | **Score** | **Ls1** | **Ls2** | **Ls3** | **Ls4** |
| --- | --- | --- | --- | --- | --- | --- | --- | --- | --- | --- |
| C(3)G | Dm | ACI96726.1 | PB314BT00336_1 | 20.67 | 0.006 | 38.5 | 7.35 | 7.11 | 7.92 | 7.89 |
| C(3)G | Dm | ACI96726.1 | PB314BT01482_1 | 21.76 | 0.025 | 36.6 | 4.45 | 8.34 | 8.31 | 3.36 |
| HIM-3 | Ce | tr\|G5EBG0\|G5EBG0_CAEEL | PB314BT01096_1 | 24.28 | 1.00E-10 | 60.5 | 12.37 | 11.99 | 11.80 | 12.25 |
| Zip1 | Sc | NP_010571.1 | PB314BT02705_1 | 22.95 | 0.002 | 40.4 | 10.85 | 12.56 | 11.94 | 11.47 |
| Hop1 | Sc | NP_012193.1 | PB314BT01096_1 | 24.02 | 8.00E-13 | 69.7 | 12.37 | 11.99 | 11.80 | 12.25 |
| SYCP1 | Mm | NP_035646.2 | PB314BT08300_1 | 21.21 | 0.055 | 35 | 7.27 | 6.24 | 5.86 | 3.66 |
| ASY2 | At | NP_194947.2 | PB314BT01096_1 | 35.44 | 2.00E-07 | 54.3 | 12.37 | 11.99 | 11.80 | 12.25 |
| ASY1 | At | NP_564896.1 | PB314BT01096_1 | 31.56 | 1.00E-26 | 113 | 12.37 | 11.99 | 11.80 | 12.25 |
| Hop1 | Sp | NP_596448.1 | PB314BT06551_1 | 55.56 | 0.012 | 37 | 12.05 | 11.54 | 11.28 | 10.89 |
| Hop1 | Sp | NP_596448.1 | PB314BT08773_1 | 33.33 | 0.012 | 37 | 12.74 | 12.98 | 12.97 | 13.42 |
| Hop1 | Sp | NP_596448.1 | PB314BT04486_1 | 27.4 | 0.046 | 35 | 14.81 | 14.07 | 13.74 | 13.94 |
| FKBP6 | Mm | sp\|Q91XW8\|FKBP6_MOUSE | PB314BT02285_1 | 41.38 | 4.00E-21 | 91.7 | 11.22 | 13.03 | 12.71 | 12.41 |
| FKBP6 | Mm | sp\|Q91XW8\|FKBP6_MOUSE | PB314BT03568_1 | 35.48 | 3.00E-17 | 76.3 | 11.36 | 9.43 | 9.18 | 9.16 |
| FKBP6 | Mm | sp\|Q91XW8\|FKBP6_MOUSE | PB314BT00177_1 | 33.98 | 2.00E-10 | 60.1 | 12.51 | 13.50 | 13.76 | 15.37 |
| FKBP6 | Mm | sp\|Q91XW8\|FKBP6_MOUSE | PB314BT07627_1 | 32.17 | 2.00E-08 | 53.9 | 11.59 | 10.35 | 10.54 | 9.32 |
| FKBP6 | Mm | sp\|Q91XW8\|FKBP6_MOUSE | PB314BT07449_1 | 36.14 | 1.00E-05 | 42.7 | 12.73 | 12.11 | 12.19 | 12.32 |
| FKBP6 | Mm | sp\|Q91XW8\|FKBP6_MOUSE | PB314BT07225_1 | 24.15 | 4.00E-04 | 40 | 9.85 | 9.76 | 10.07 | 11.67 |
| FKBP6 | Mm | sp\|Q91XW8\|FKBP6_MOUSE | PB314BT05286_1 | 35.29 | 0.016 | 34.7 | 12.33 | 12.69 | 12.78 | 13.57 |
| FKBP6 | Mm | sp\|Q91XW8\|FKBP6_MOUSE | PB314BT00046_1 | 35.48 | 0.037 | 34.3 | 12.12 | 11.65 | 11.71 | 12.48 |
| SYP-2 | Ce | XP_002641990.1 | PB314BT04320_1 | 24.74 | 0.048 | 33.1 | 9.11 | 8.88 | 8.93 | 5.02 |
| REC8 | Os | AY371049 | PB314BT03649_1 | 29.7 | 1.00E-12 | 68.6 | 13.81 | 12.06 | 12.66 | 13.86 |
| REC8 | Os | AY371049 | PB314BT00749_1 | 24.79 | 1.00E-11 | 65.9 | 10.24 | 11.34 | 11.86 | 13.42 |
| MER3 (RMC1) | Os | FJ008126 | PB314BT02265_1 | 34.41 | 2.00E-133 | 434 | 14.71 | 14.26 | 13.58 | 13.38 |
| MER3 (RMC1) | Os | FJ008126 | PB314BT03845_1 | 31.44 | 1.00E-85 | 306 | 14.86 | 14.69 | 14.06 | 14.32 |
| MER3 (RMC1) | Os | FJ008126 | PB314BT08755_1 | 31.01 | 1.00E-82 | 297 | 14.99 | 14.48 | 14.18 | 14.00 |
| PAIR2 (OsASY1) | Os | AB109238 | PB314BT01096_1 | 32.58 | 8.00E-30 | 123 | 12.37 | 11.99 | 11.80 | 12.25 |
| ZYP1a | At | NM_102076 | PB314BT02216_1 | 51.61 | 0.42 | 32.7 | 7.28 | 11.60 | 11.45 | 12.13 |
| SPO11 | At | AT1G63990.1 | PB314BT06505_1 | 29.96 | 6.00E-32 | 123 | 9.08 | 10.48 | 11.25 | 13.04 |
| MSH5 | At | AT3G20475.1 | PB314BT05870_1 | 31.67 | 4.00E-104 | 355 | 12.77 | 13.13 | 13.31 | 14.98 |
| MSH4 | At | AT4G17380.1 | PB314BT01474_1 | 35.18 | 1.00E-124 | 397 | 11.30 | 12.05 | 11.95 | 12.39 |
| MND1 | At | AT4G29170.1 | PB314BT02066_1 | 33.33 | 9.00E-34 | 122 | 11.19 | 11.15 | 11.12 | 12.56 |

a = For protein description, see^9^.

b = Dm = *Drosophila melanogaster*, Sp = *Schizosaccharomyces pombe,* Mm = *Mus musculus*, Ce = *Caenorhabditis elegans,* At = *Arabidopsis thaliana,* Os *= Oryzae sativa.*

**Supplementary Table S5. Sequence similarity search with the *P. brassicae* e3 mitochondrial sequence.** BLASTn search of the whole-genome shotgun sequence (WGS) database with the *P. brassicae* e3 mitochondrial sequence as a query.

| **Best BLASTn hit^a^** | **E-value** | **Query cover %^b^** | **Ident. %** | **Accession no.** |
| --- | --- | --- | --- | --- |
| *P. brassicae* Pldbra_eH_r1s043^c^ | 0.0 | 97 | 100 | POCA01000043.1 |
| *P. brassicae* ZJ-1 scaffold49^d^ | 0.0 | 80 | 99.99 | MCBL01000050.1 |
| *P. brassicae* AAFC-SK-Pb3 PbPT3Sc00063^e^ | 0.0 | 81 | 99.95 | RZOB01000060.1 |

a = WGS search was limited to Plasmodiophorida (taxid:37357) and included 4,894 sequences (March 2019).

b = e3 mitochondrial sequence (GenBank LS992577) with 114,663 bp was used as a query.

c = eH strain mitochondrial sequence with 102,962 bp^5^

d = ZJ-1 strain mitochondrial sequence with 93,640 bp^6^

e = Pb3 strain mitochondrial sequence with 101,023 bp^7^

**Supplementary Table S6.** **Mitochondrial sequence from *P. brassicae* e3 strain earlier generated by Illumina/454 technology^2^** (Excel file).

**Supplementary Table S7. Comparison between *P. brassicae* e3 and *Spongospora subterranea* mitochondrial genomes.**

| **General features** | ***P. brassicae* e3** | ***S. subterranea***^10^ |
| --- | --- | --- |
| Genome size (bp) | 114,663 | 37,699 |
| Genome shape | Circular | Circular |
| Overall GC content % | 26.2 | 26.8 |
| Simple repeats % | 1.5^a^ | NA |
| Coding density (coding bp/total bp) % | 54 | 66 |
| **Gene content** |  |  |
| Respiratory chain protein-coding genes | 17 (12)**^b^** | 16 (3)**^b^** |
| Ribosomal proteins | 14 (4 *rpl* and 10 *rps*) | 12 (3 *rpl* and 9 *rps*) |
| RNA-directed DNA polymerase | 1 (*rdp*) | 1 (*rdp*) |
| Ribosomal RNA genes | 3 (*rnl*, *rns* and *rrn5*) | 3 (*rnl*, *rns* and *rrn5*) |
| Transfer RNA genes | 24^c^ | 24^c^ |
| RNA processing genes | 1 (*rnpB*) | 1 (*rnpB*) |
| ORFs with functional information | 10 (3 intergenic and 7 within introns)^d^ | orf22^e^ |
| Hypothetical ORFs | 9 (4 intergenic and 5 within introns)^d^ | 2 (intergenic) |
| Total number | 79 | 59 |

a = List of *P. brassicae* e3 simple repeats in Supplementary Table S12.

b = Number of protein-coding genes containing introns is indicated in brackets.

c = List of transfer RNA genes in Supplementary Table S9.

d = List of *P. brassicae* e3 ORFs and functional information in Supplementary Table S10.

e = orf22 was functionally annotated as *rdp* gene.

**Supplementary Table S8. Comparison of protein-coding genes in mitochondrial genomes**

**of *P. brassicae* e3 and *Spongospora subterranea.***

◼= presence, ☐ = absence in database. Number of introns is indicated in brackets.

| **Protein genes** | ***P. brassicae* e3** | ***S. subterranea*^10,a^** |
| --- | --- | --- |
| *atp1* | ◼ (3) | ◼ |
| *atp4* | ☐ | ☐ |
| *atp6* | ◼ (1) | ◼ |
| *atp8* | ◼ | ☐ |
| *atp9* | ◼ | ◼ |
| *cob* | ◼ (5) | ◼ (2) |
| *cox1* | ◼ (13) | ◼ |
| *cox2* | ◼ (1) | ◼ |
| *cox3* | ◼ (2) | ◼ |
| *nad1* | ◼ (3) | ◼ (2) |
| *nad2* | ◼ | ◼ |
| *nad3* | ◼ | ◼ (1) |
| *nad4* | ◼ (1) | ◼ |
| *nad4L* | ◼ | ◼ |
| *nad5* | ◼ (4) | ◼ |
| *nad6* | ◼ (1) | ◼ |
| *nad7* | ◼ (6) | ◼ |
| *nad9* | ◼ (1) | ◼ |
| *rdp* | ◼ | ◼ |
| *rpl2* | ☐ | ☐ |
| *rpl5* | ◼ | ☐ |
| *rpl6* | ◼ | ◼ |
| *rpl14* | ◼ | ◼ |
| *rpl16* | ◼ | ◼ |
| *rps3* | ◼ | ◼ |
| *rps4* | ◼ | ◼ |
| *rps7* | ◼ | ◼ |
| *rps8* | ◼ | ◼ |
| *rps10* | ◼ | ☐ |
| *rps11* | ◼ | ◼ |
| *rps12* | ◼ | ◼ |
| *rps13* | ◼ | ◼ |
| *rps14* | ◼ | ◼ |
| *rps19* | ◼ | ◼ |

a = In the S. *subterranea* genome the *rdp* gene was mistakenly assigned as “*rdrp*” in Figure 1.

The *rdp* sequence (GenBank AIK19930.1) represents the correct entry (personal communication with P. Gutiérrez).

**Supplementary Table S9.** **Comparison of structural RNA genes in**

**mitochondrial genomes of *P. brassicae* e3 and *Spongospora subterranea*.**

Number of tRNA copies with the same anti-codon indicated in brackets.

◼ = presence, ☐ = absence.

| **RNA genes** | ***P. brassicae* e3^a^** | ***S. subterranea*^b^** |
| --- | --- | --- |
| *rnl* | ◼ 8^c^ | ◼ |
| *rns* | ◼ 5^c^ | ◼ |
| *rrn5* | ◼ | ◼ |
| *rnpB* | ◼ | ◼ |
| *trnA* | TGC | TGC |
| *trnC* | GCA | GCA |
| *trnD* | GTC | GTC |
| *trnE* | TTC | TTC |
| *trnF* | GAA | GAA |
| *trnG* | TCC | TCC |
| *trnH* | GTG | GTG (x2) |
| *trnI* | GAT | GAT |
| *trnK* | TTT | TTT |
| *trnL* | ☐ | ☐ |
| *trnL* | TAA | TAA |
| *trnL* | TAG | TAG |
| *trnM* | CAT (x2) | CAT (x2) |
| *trnN* | GTT | GTT |
| *trnP* | TGG | TGG |
| *trnQ* | TTG | ☐ |
| *trnR* | TCG | TCG |
| *trnR* | TCT | TCT |
| *trnS* | GCT | GCT |
| *trnS* | TGA | TGA |
| *trnT* | TGT | TGT |
| *trnV* | TAC | TAC |
| *trnW* | ☐ | ☐ |
| *trnW* | TCA | TCA |
| *trnY* | GTA | GTA |

a = tRNA anti-codons annotated by tRNAscan-SE. U replaced with T for easier comparison.

b = tRNA anti-codons annotated by tRNAscan-SE in the S. *subterranea* mitochondrial sequence (GenBank KF738139).

c = No. of introns in ribosomal RNAs.

**Supplementary Table S10. Information about ORFs in the *P. brassicae* e3 mitochondrial genome.** Functional information retrieved by sequence similarity searches of the Uniprot/Swiss-Prot database with ORF sequences as queries and a maximal e-value of 10e-6.

| **ORF** | **Region^a^** | **Length (aa)** | **Best hit^b, c^** | **Accession no.** | **Organism^d^** |
| --- | --- | --- | --- | --- | --- |
| ORF511 | *nad7*-I4 | 511 | No hits ≤ e-value 10e-6 |  |  |
| ORF505 | *nad7*-I3 | 505 | Maturase-like protein 1(*mat*1)^c^ | P05728 | *E. gracilis* |
| ORF61 | *atp6*-I1 | 499 | No hits ≤ e-value 10e-6 |  |  |
| ORF688 | *nad6*-I1 | 688 | DNA-directed RNA polymerase (*rpoC*2)^c^ | Q25802 | *P. falciparum* |
| ORF465 | *nad5*-I1 | 465 | Ribosomal operon-associated A (*roaA*)^c^ | P58145 | *E. longa* |
| ORF395 | intergenic | 395 | No hits ≤ e-value 10e-6 |  |  |
| ORF1512 | intergenic | 1512 | Thrombospondin motifs (*gon*-1)^c^ | Q19791 | *C. elegans* |
| ORF1164 | intergenic | 1164 | No hits ≤ e-value 10e-6 |  |  |
| ORF740 | intergenic | 764 | Proline-rich protein (*PRR*36)^c^ | Q9H6K5 | *H. sapiens* |
| ORF27 | intergenic | 241 | No hits ≤ e-value 10e-6 |  |  |
| ORF750^e^ | *cob*-I1 | 750 | Maturase-like protein 2 (*mat*2)^b^ | P31916 | *E. gracilis* |
| ORF114 | *cob*-I4 | 353 | No hits ≤ e-value 10e-6 |  |  |
| ORF504 | intergenic | 504 | DNA-directed RNA polymerase (*rpoB*)^c^ | Q9NJN5 | *N. caninum* |
| ORF580^e^ | *cox1*-I4 | 580 | Group II intron-encoded protein (*LtrA*) ^b^ | P0A3U1 | *L. lactis* |
| ORF117 | *cox1*-I7 | 550 | No hits ≤ e-value 10e-6 |  |  |
| ORF118 | *cox1*-I8 | 676 | Maturase-like protein 2 (*mat*2)^c^ | P31916 | *E. gracilis* |
| ORF110 | *cox1*-I10 | 385 | No hits ≤ e-value 10e-6 |  |  |
| ORF671^e^ | *cox1*-I12 | 671 | DNA binding endonuclease (*spmit.*06)^b^ | P05511 | *S. pombe* |
| ORF621 | intergenic | 280 | No hits ≤ e-value 10e-6 |  |  |

a = I indicates intron.

b = BLASTp search of the UniProt/Swiss-Prot reference data set with 551,705 protein sequences.

c = Ssearch of the UniProt/Swiss-Prot reference data set with 559,228 protein sequences.

d = *Euglena gracilis, Plasmodium falciparum, Euglena longa, Caenorhabditis elegans,* *Homo sapiens, Neospora caninum, Lactococcus lactis, Schizosaccharomyces pombe.*

e = ORF750 was re-named to *mat*2, ORF580 to *LtrA* and ORF671 to *spmit.*06 in the ENA submitted data and Figure 3a.

**Supplementary Table S11. Intron group II and transposon-like elements in the *P. brassicae* e3 mitochondrial genome.**

| **Category** | **Start bp** | **End bp** | **Size (nt)** | **Region** |
| --- | --- | --- | --- | --- |
| Intron group II | 2592 | 2666 | 75 | *rnl*, in proximity of intron5-exon6 boundary |
| Long Terminal Repeat | 3610 | 3935 | 326 | *rnl*, spanning intron3-exon4-intron4 boundaries |
| Intron group II | 19222 | 19288 | 67 | *nad7*, in proximity of intron3-exon4 boundary |
| Intron group II | 27702 | 27767 | 66 | *atp6*, in proximity of intron1-exon2 boundary |
| Intron group II | 29556 | 29657 | 102 | *nad6*, spanning intron1-exon2 boundary |
| Intron group II | 35281 | 35391 | 111 | *nad5*, spanning intron2-exon3 boundary |
| Enhancer/Suppressor-mutator | 47283 | 49844 | 2562 | Intergenic region |
| Intron group II | 91247 | 91361 | 115 | *cox1*, spanning intron9-exon10 boundary |
| Intron group II | 103079 | 103193 | 115 | *cox2*, spanning intron1-exon2 boundary |

**Supplementary Table S12. Simple repeats in the *P. brassicae* e3**

**mitochondrial genome.**

| **Repeat** | **Start bp** | **End bp** |
| --- | --- | --- |
| (TA)_n_ Simple repeat | 8887 | 8930 |
| (TATATT)_n_ Simple repeat | 10019 | 10069 |
| A-rich Low complexity | 11993 | 12040 |
| A-rich Low complexity | 12281 | 12307 |
| A-rich Low complexity | 13543 | 13591 |
| (AATAA)_n_ Simple repeat | 14670 | 14713 |
| (AATATT)_n_ Simple repeat | 16181 | 16213 |
| (AT)_n_ Simple repeat | 19732 | 19771 |
| (AATAA)_n_ Simple repeat | 20220 | 20264 |
| (TATTG)_n_ Simple repeat | 21216 | 21248 |
| (TTTCT)_n_ Simple repeat | 24874 | 24900 |
| A-rich Low complexity | 26172 | 26227 |
| (TTTATTA)_n_ Simple repeat | 28231 | 28308 |
| (AATA)_n_ Simple repeat | 33716 | 33742 |
| (ATTTAA)_n_ Simple repeat | 57715 | 57754 |
| (T)_n_ Simple repeat | 58171 | 58201 |
| (TTATT)_n_ Simple repeat | 60956 | 61010 |
| (TTAAA)_n_ Simple repeat | 62110 | 62137 |
| (ATAA)_n_ Simple repeat | 67463 | 67504 |
| (T)_n_ Simple repeat | 71282 | 71300 |
| (TATT)_n_ Simple repeat | 71925 | 71956 |
| (T)_n_ Simple repeat | 72734 | 72762 |
| (TTATT)_n_ Simple repeat | 74054 | 74112 |
| (TTATT)n Simple repeat | 76321 | 76366 |
| (ATT)_n_ Simple repeat | 77072 | 77139 |
| (ATT)_n_ Simple repeat | 79736 | 79775 |
| (TTTAA)_n_ Simple repeat | 85502 | 85530 |
| mitc (TTTTA)_n_ Simple repeat | 93618 | 93686 |
| (TTTTAT)_n_ Simple repeat | 94669 | 94700 |
| (GTTATTT)_n_ Simple repeat | 96066 | 96100 |
| (ATTAT)_n_ Simple repeat | 96703 | 96754 |
| (ATTT)_n_ Simple repeat | 99770 | 99797 |
| (T)_n_ Simple repeat | 100340 | 100375 |
| (TAATTT)_n_ Simple repeat | 101503 | 101534 |
| (ATTAGTA)_n_ Simple repeat | 105475 | 105549 |
| A-rich Low complexity | 105680 | 105764 |
| (T)_n_ Simple repeat | 106285 | 106308 |
| A-rich Low complexity | 110279 | 110309 |
| (ATATTA)_n_ Simple repeat | 110747 | 110778 |
| (ATTAT)_n_ Simple repeat | 110881 | 110960 |
| (TAA)_n_ Simple repeat | 113269 | 113312 |

**Supplementary Table S13. Start and stop codons of protein-coding genes**

**in the *P. brassicae* e3 mitochondrial genome.**

| **Protein genes** | **Start codon** | **Amino acid** | **Stop codon** |
| --- | --- | --- | --- |
| *atp1* | ATG | Methionine | TAA |
| *atp6* | TTA^a^ | Leucine | TAA |
| *atp8* | ATG | Methionine | TAA |
| *atp9* | ATG | Methionine | TAG |
| *cob* | ATG | Methionine | TAG |
| *cox1* | TTA^a^ | Leucine | TAA |
| *cox2* | ATG | Methionine | TAA |
| *cox3* | TTA^a^ | Leucine | TAA |
| *nad1* | ATG | Methionine | TAA |
| *nad2* | ATG | Methionine | TAA |
| *nad3* | ATC^a^ | Isoleucine | TAA |
| *nad4* | ATT^a^ | Isoleucine | TAG |
| *nad4L* | ATG | Methionine | TAG |
| *nad5* | GTG^a^ | Valine | TAA |
| *nad6* | ATG | Methionine | TAG |
| *nad7* | ATG | Methionine | TAA |
| *nad9* | ATG | Methionine | TAA |
| *rdp* | ATG | Methionine | TAA |
| *rpl5* | ATG | Methionine | TAA |
| *rpl6* | TTG^a^ | Leucine | TAA |
| *rpl14* | ATG | Methionine | TAG |
| *rpl16* | ATG | Methionine | TAA |
| *rps3* | ATG | Methionine | TAA |
| *rps4* | ATG | Methionine | TAA |
| *rps7* | ATG | Methionine | TAG |
| *rps8* | ATG | Methionine | TAA |
| *rps10* | ATG | Methionine | TAG |
| *rps11* | ATG | Methionine | TAA |
| *rps12* | ATG | Methionine | TAA |
| *rps13* | ATG | Methionine | TAA |
| *rps14* | TTG^a^ | Leucine | TAA |
| *rps19* | ATT^a^ | Isoleucine | TAA |

a = Alternative codon commonly translated into methionine.

**Supplementary Table S14. Group II introns annotated by**

**the MFannot tool in the *P. brassicae* e3 mitochondrial genome.**

The MFannot^11^ annotations were not uploaded to the ENA database since

no support from any other annotation tools was achieved.

| **Start bp** | **End bp** | **Region^a^** |
| --- | --- | --- |
| 732 | 778 | *rnl* |
| 2619 | 2664 | *rnl* |
| 5083 | 5123 | *rnl* |
| 8333 | 8377 | *rns* |
| 9603 | 9648 | *rns* |
| 12975 | 13486 | *nad4*-I1 |
| 14036 | 14080 | Intergenic *nad4*-*nad7* |
| 14863 | 15622 | *nad7*-I6 |
| 17040 | 19154 | *nad7*-I4 |
| 19222 | 21606 | *nad7*-I3 |
| 21733 | 22386 | *nad7*-I2 |
| 22531 | 23160 | *nad7*-I1 |
| 25580 | 27763 | *atp6*-I1 |
| 28789 | 28831 | Intergenic *atp6*-*nad3* |
| 29649 | 29655 | *nad6*-I1 |
| 34222 | 34893 | *nad5*-I3 |
| 35367 | 35389 | *nad5*-I2 |
| 35902 | 35945 | *nad5*-I2 |
| 36466 | 36509 | *nad5*-I1 |
| 62439 | 62829 | *cob*-I1 |
| 65488 | 66255 | *cob*-I2 |
| 67174 | 67614 | *cob*-I4 |
| 70497 | 70539 | Intergenic *cob*-*nad1* |
| 76382 | 76426 | Intergenic orf504-*nad4L* |
| 79313 | 79358 | Intergenic *rdp*-*cox1* |
| 79802 | 79843 | Intergenic *rdp*-*cox1* |
| 81203 | 81245 | *cox1*-I2 |
| 85245 | 85806 | *cox1*-I6 |
| 85858 | 87839 | *cox1*-I7 |
| 87944 | 90588 | *cox1*-I8 |
| 90632 | 91314 | *cox1*-I9 |
| 91375 | 93802 | *cox1*-I10 |
| 94777 | 97798 | *cox1*-I12 |
| 97844 | 98602 | *cox1*-I13 |
| 103081 | 103127 | *cox2* |
| 104052 | 104094 | Intergenic *cox2*-*nad9* |
| 105174 | 105219 | *nad9* |
| 105876 | 105919 | Intergenic *nad9*-*cox3* |
| 109198 | 109640 | *cox3*-I2 |
| 109752 | 110657 | Intergenic *cox3*-*atp1* |
| 111852 | 112317 | *atp1-*I1 |
| 112474 | 113013 | *atp1*-I2 |
| 113152 | 113798 | *atp1*-I3 |

a = I indicates intron.

**Supplementary Table S15.** **Information on organisms and protein-coding genes included in phylogenetic analyses** (Excel file).

**Supplementary Note**

**Materials and sequencing**

Resting spores from clubs of *Brassica rapa* cv. Granaat grown in *Plasmodiophora brassicae* strain e3 infested soils were isolated and used for DNA extraction^12^ and later on purity check^2^. Approximately 230 µg high-quality DNA was sent to SciLifeLab, Uppsala, Sweden for PacBio RSII sequencing. For PacBio library construction, genomic DNA was sheared to 10 kb and converted into the SMRTbell library format using an RS DNA Template Preparation Kit. SMRTbell templates were subjected to standard SMRT sequencing on the PacBio RS system according to the manufacturer’s protocol.

**Nuclear assembly and annotation**

Raw reads were assembled using FALCON v0.4^13^ and HAGP3 from the SMRTportal v2.3 (PacBio, Menlo Park, CA, USA). The two assemblies were manually merged and polished using Quiver from SMRTportal v2.3. The gene annotation pipeline MAKER v2.3^14^ was used in combination with *ab-initio* gene predictors: Augustus v2.5.5^15^, SNAP^16^ and GeneMark-ES v2.3^17^. Augusts and SNAP were trained on the previously annotated and manually curated *P. brassicae* nuclear genes^2^. The UniProt/Swiss-Prot database and all rhizarian ESTs and proteins found at NCBI, as well as transcripts assembled from strand-specific RNAseq *P. brassicae* libraries^2^ were used as evidence integrated in gene predictions. Annotation of repeats was performed within MAKER, using a *P. brassicae* specific repeat library constructed *de novo* using RepeatModeler v1.0.7^18^ as well as MAKER’s internal library of transposable elements and the Repbase repeat library rm-20130422. MAKER was run using default parameters except *pred_flank* that was set to 100 bp, *split_hit*. Telomeric repeats were identified by using Tandem Repeats Finder^19^. For biological pathway classifications we used the WebMGA and the KOG classification tools^20^. Additional protein analyses were done using: OrthoMCL^1^, sequence similarity searches, BLASTP searches against GenBank non-redundant protein database, HMM-searches against Pfam database, and RPS-BLAST searches against NCBI KOG (March 2017).

**Mitochondrial genome *de novo* assembly and annotation**

The contig encoding the mitochondrial genome was assembled using Canu v1.5^21^. Visualization of the raw assembly with Bandage^22^ generated a circular contig (133,222 bp) in which overlapping sequences were identified by Gepard v1.40^4^. After removing overlaps (18,559 bp) and circularization, the final 114,663 bp long PacBio sequence was polished using Quiver. Mapping of the Illumina data^2^ to the PacBio sequence using BWA v0.7.15^23^ and SAMtools v1.5^24^ and polishing with Pilon^25^, revealed 2 x 1 bp difference, which were corrected. The coverage was further checked by aligning the PacBio generated reads to the assembled mitochondrial genome using the GraphMap v0.5.2^26^ tool. To optimize *de novo* annotation of the PacBio mitochondrial sequence several tools and sources were used and combined with manual curation. Automated annotations were done using MFannot v1.33^11^ with the genetic code 4 “Mold, protozoan and coelenterate mitochondrial; Mycoplasma/Spiroplasma”, and also Prokka v1.1^27^ with mitochondria and archaea kingdoms and MAKER2^28^ using Repeatmasker^29^, tRNAscan-SE^30^, Uniprot/Swiss-Prot mitochondrial proteins (Nov. 2016), the ribosomal database^31^ and Rfam v12^32^. Further information was provided by transcriptome data from^2^ re-assembled using Trimmomatic^33^, Tophat^34^ and Stringtie^35^. The *S. subterranea* annotated mitochondrial genome^10^ (GeneBank accession KF738139) was used as an additional source. Based on the different lines of annotation and sources, the gene models have been manually created through Web Apollo^36^. Translated CDS features were blasted against the Uniprot/Swiss-Prot reference data set (Aug. 2016) and filtered using a maximal e-value of 10e-6 and run against InterProScan v5.21-60^37^. All retrieved functional information have been integrated into the final annotated data set. Predicted ORFs were used as query sequences for sequence similarity searches of the Uniprot/Swiss-Prot database (March 2019) and domain prediction with InterProScan v5.34-73.0^38^.

**Mitochondrial sequence comparison**

Dot-plots created by Gepard v1.40^4^ were used for comparison of *P. brassicae* mitochondrial sequences from four strains: e3 (generated in this study, GeneBank accession LS992577), eH^5^ (GeneBank accession POCA01000043), ZJ-1^6^ (GeneBank accession MCBL01000050) and the Pb3 strain^7^ (GeneBank accession RZOB01000060). Additional sequence comparison was done using two mitochondrial contigs of the e3 strain generated by Illumina/454 sequencing but excluded from^2^. The data is now provided in Supplementary Table S6. Synteny between the mitochondrial sequences from four *P. brassicae* strains and *S. subterranea*^10^ (GeneBank accession KF738139) was tested using the Mauve genome alignment tool v2.4.0^8^ with default settings.

**Phylogenetic analysis**

Amino acid sequences were retrieved from public databases for 12 mitochondrial protein-coding genes (*cob*, *cox1*, *cox2*, *cox3*, *atp6*, *nad1*, *nad2*, *nad3*, *nad4*, *nad4L, nad5* and *nad6*) conserved across 67 organisms. The 63 organisms were selected to represent major eukaryotic groups with available complete mitochondrial genomes and if possible, deep-branching positions. The 4 α-protebacteria were selected as outgroup species. The sequences were aligned using Clustal Omega v1.2.1^39^ with default setting. Multiple alignments were visualized with AliView^40^, examined and automatically trimmed with trimAL v1.4^41^. Maximum likelihood (ML) phylogenetic trees with rapid bootstrap (RB) analyses were generated with RAxML v8.2.11^42^. The best amino acid substitution model was estimated with PROTGAMMAUTO option for each single gene tree and run with 250 to 650 RB, a number of iterations predicted to be sufficient by the autoFC stopping criteria. For concatenated trees, 12 protein alignments were concatenated (the script is available at https://github.com/nylander/catfasta2phyml) into a super-matrix comprising 773 genes and 3,819 aligned amino acid positions. ML analyses were inferred under GAMMA rate of heterogeneity with the substitution models specified for each partition and run with 250 RB iterations. Trees were displayed and edited with Dendroscope v3.5.9^43^. Information on organisms and proteins and substitution models are listed in Supplementary Table S15.

**Supplementary References**

1. Li, L., Stoeckert, C.J., Jr. & Roos, D.S. OrthoMCL: identification of ortholog groups for eukaryotic genomes. *Genome Res.***13**, 2178–2189 (2003).

2. Schwelm, A. *et al.* The *Plasmodiophora brassicae* genome reveals insights in its life cycle and ancestry of chitin synthases. *Sci. Rep.* **5**, 11153 (2015).

3. Keeling, P.J. *et al*. The marine microbial eukaryote transcriptome sequencing project (MMETSP): illuminating the function a diversity of eukaryotic life in the oceans through transcriptome sequencing. *PLoS Biol.* **12**, e1001889 (2014).

4. Krumsiek, J., Arnold, R. & Rattei, T. Gepard: a rapid and sensitive tool for creating dotplots on genome scale, Bioinformatics **23**, 1026–1028 (2007).

5. Daval, S. *et al.* Computational analysis of the *Plasmodiophora brassicae* genome: mitochondrial sequence description and metabolic pathway database design. *Genomics* <https://doi.org/10.1016/j.ygeno.2018.11.013>.

6. Bi, K. *et al.* Integrated omics study of lipid droplets from *Plasmodiophora brassicae*. *Scientific Rep.* **6**, 36965 (2016).

7. Rolfe, S.A. *et al.* The compact genome of the plant pathogen *Plasmodiophora brassicae* is adapted to intracellular interactions with host *Brassica* spp. *BMC Genom.* **17**, 1–15 (2016).

8. Darling, A.C.E., Mau, B., Blattner, F.R. & Perna, N.T. Mauve: Multiple alignment of conserved genomic sequence with rearrangements. *Genome Res.* **14**,1394-1403 (2004).

9. Grishaeva, T.M. & Bogdanov, Y.F. Conservation and variability of synaptonemal complex proteins in phylogenesis of eukaryotes. *Int. J. Evol. Biol.* **2014**, 856230 (2014).

10. Gutiérrez, P., Bulman, S., Alzate, J., Ortíz, M.C. & Marin, M. Mitochondrial genome sequence of the potato powdery scab pathogen *Spongospora subterranea*. *Mitochondrial DNA Part A* **27**, 58-59 (2016).

11. Beck, N. & Lang, B.F. MFannot, organelle genome annotation webserver. <http://megasun.bch.umontreal.ca/cgi-bin/mfannot/mfannotInterface.pl> (2010).

12. Mehrabi, S., Stjelja, S. & Dixelius, C. Disease establishment, resting spore isolation and DNA extraction of *Plasmodiophora brassicae*, the clubroot pathogen. *Bio-Protocol* **101,** e2864 (2018).

13. Chin, C.S. *et al*. Phased diploid genome assembly with single molecule real-time sequencing. *Nat. Meth.* **13**, 1050–1054 (2016).

14. Cantarel, B.L. *et al.* MAKER: An easy-to-use annotation pipeline designed for emerging model organism genomes. *Genome Res.* 1**8,** 188–196 (2008).

15. Stanke, M., Steinkamp, R., Waack, S. & Morgenstern, B. AUGUSTUS: a web server for gene finding in eukaryotes. *Nucleic Acids Res.* **32**, W309–312 (2004).

16. Korf, I. Gene finding in novel genomes. *BMC Bioinformatics* **5**, 59 (2004).

17. Ter-Hovhannisyan, V., Lomsadze, A., Chernoff, Y. & Borodovsky, M. Gene prediction in novel fungal genomes using an ab initio algorithm with unsupervised training. *Genome Res.* **18**, 1979–1990 (2008).

18. Smit, A.F.A. & Hubley, R. RepeatModeler Open-1.0. [http://www.repeatmasker.org](http://www.repeatmasker.org/) (2008-2015).

19. Benson, G. Tandem repeats finder: a program to analyze DNA sequences. *Nucleic Acids Res.* **27,** 573-580 (1999).

20. Wu, S., Zhu, Z., Fu, L., Niu, B. & Li, W. WebMGA: a Customizable Web Server for Fast Metagenomic Sequence Analysis. *BMC Genom.* **12**, 444 (2011).

21. Koren, S. *et al*. Canu: scalable and accurate long-read assembly via adaptive *k*-mer weighting and repeat separation. *Genome Res.* **27**,722-736 (2017).

22. Wick, R.R., Schultz, M.B., Zobel, J. & Holt, K.E. Bandage: interactive visualization of de novo genome assemblies, Bioinformatics **31**, 3350–3352 (2015).

23. Li, H. Aligning sequence reads, clone sequences and assembly contigs with BWA-MEM. *arXiv*:1303.3997 [q-bio.GN] (2013).

24. Li, H. *et al.* 1000 Genome Project Data Processing Subgroup. The sequence alignment/Map format and SAMtools. *Bioinformatics* **25**, 2078-2079 (2009).

25. Walker, B.J. *et al.* Pilon: an integrated tool for comprehensive microbial variant detection and genome assembly improvement. *PLoS One* **9,** e112963 (2014).

26. Sović, I. *et al.* Fast and sensitive mapping of nanopore sequencing reads with GraphMap. *Nat. Com*. **7,** 11307 (2016).

27. Seemann, T. Prokka: rapid prokaryotic genome annotation. *Bioinformatics* **30**, 2068–2069 (2014).

28. Holt, C. & Yandell, M. MAKER2: an annotation pipeline and genome-database management tool for second-generation genome projects. *BMC Bioinformatics* **12**, 491 (2011).

29. Smit, A.F.A., Hubley, R. & Green, P. RepeatMasker Open-3.0. <http://www.repeatmasker.org> (2010).

30. Schattner, P., Brooks, A.N. & Lowe, T.M. The tRNAscan-SE, snoScan and snoGPS web servers for the detection of tRNAs and snoRNAs. *Nucleic Acids Res.* **33**, 686–689 (2005).

31. Cole, J.R. *et al.* Ribosomal Database Project: Data and tools for high throughput rRNA analysis. *Nucleic Acids Res.* **42**, 633–642 (2014).

32. Nawrocki, E.P. Rfam 12.0: updates to the RNA families database. *Nucleic Acids Res.* **43**, D130-137 (2015).

33. Bolger, A.M., Lohse, M. & Usadel, B. Trimmomatic: a flexible trimmer for Illumina sequence data. *Bioinformatics* **30**, 2114-2120 (2014).

34. Kim, D. *et al*. TopHat2: accurate alignment of transcriptomes in the presence of insertions, deletions and gene fusions. *Genome Biol.* **14**, R36 (2013).

35. Pertea, M. *et al.* StringTie enables improved reconstruction of a transcriptome from RNA seq reads. *Nature Biotechnol.* **33**, 290-295 (2015).

36. Lee, E. *et al.* Web Apollo: a web-based genomic annotation editing platform. *Genome Biol.* **14**, R93 (2013).

37. Jones, P. *et al.* InterProScan 5: genome-scale protein function classification. *Bioinformatics* **30**,1236–1240 (2014).

38. Mitchell, A.L. *et al*. InterPro in 2019: improving coverage, classification and access to protein sequence annotations. *Nucleic Acids Res.* **47**, D351-D360 (2019).

39. Sievers, F. *et al*. Fast, scalable generation of high-quality protein multiple sequence alignments using Clustal Omega. *Mol. Systems Biol.* **7**, 539 (2011).

40. Larsson, A. AliView: a fast and lightweight alignment viewer and editor for large data sets. *Bioinformatics* **30**, 3276-3278 (2014).

41. Capella-Gutiérrez, S., Silla-Martínez, J.M. & Gabaldón, T. trimAl: a tool for automated alignment trimming in large-scale phylogenetic analyses, Bioinformatics **25**, 1972–1973 (2009).

42. Stamatakis, A. RAxML version 8: A tool for phylogenetic analysis and post-analysis of large phylogenies. *Bioinformatics* **30**, 1312-1313 (2014).

43. Huson, D. & Scornavacca, C. Dendroscope 3: An interactive tool for rooted phylogenetic trees and networks. *System. Biol.* **61**, 1061–1067 (2012).
